# Detection and characterization of antiviral-resistant viruses during the influenza season of 2024–25

**DOI:** 10.1128/spectrum.01514-26

**Published:** 2026-08-06

**Authors:** Mira C. Patel, Ha T. Nguyen, Philippe Noriel Q. Pascua, Mercedes Lopez-Esteva, Chloe Champion, Vasiliy P. Mishin, Han Di, Kristine Lacek, Marie K. Kirby, Allen Bateman, Maureen Sullivan, Susan Trow, Jennifer Laplante, Kirsten St. George, Benjamin Rambo-Martin, Angiezel Merced-Morales, Alicia Budd, Rebecca J. Kondor, Charles T. Davis, Larisa V. Gubareva

**Affiliations:** 1 Influenza Division, National Center for Immunization and Respiratory Diseases, Centers for Disease Control and Prevention 209774, Atlanta, Georgia, USA; 2 Chippewa Government Solutions, Sault Tribe Incorporated 159923 , Marie, Michigan, USA; 3 Wisconsin State Laboratory of Hygiene, University of Wisconsin-Madison 37360, Madison, Wisconsin, USA; 4 Association of Public Health Laboratories 8282, Bethesda, Maryland, USA; 5 Laboratory of Viral Diseases, Wadsworth Center, New York State Department of Health 1094, Albany, New York, USA; Erasmus MC, Rotterdam, the Netherlands

**Keywords:** neuraminidase inhibitor, baloxavir, oseltamivir, antiviral resistance, influenza, reduced susceptibility, WGS

## Abstract

During the high severity season of 2024–25, CDC with public health partners sequenced and analyzed genomes of >10,000 influenza viruses for antiviral resistance markers. Available sequence-flagged and representative viruses were tested with antivirals using *in vitro* assays. In the US, three oseltamivir-resistant A(H3N2) viruses had treatment-emergent neuraminidase (NA) mutations, either E119V or R292K. Oseltamivir-resistant A(H1N1)pdm09 viruses with NA-H275Y were detected in 15 states, albeit at a low frequency (0.53%). They belonged to several phylogenetic groups, with hemagglutinin (HA) subclade D.3.1 combined with either NA subclade D.1 or D.2 being most common. Based on shared sequence data, nearly all H275Y viruses from Australia, Canada, and Chile also belonged to these HA and NA subclades. Conversely, most H275Y viruses (68/81) from China belonged to HA subclade C.1.9 and NA subclade D and shared the permissive mutation R257K. Influenza polymerase acidic (PA) mutations conferring 4- to 92-fold decreased baloxavir susceptibility were detected in nine influenza A viruses. Viruses with PA-I38T showed mild attenuation of replicative fitness in three cell lines. Based on available data, NA-H275Y and PA-I38T viruses were collected from patients with no exposure to antivirals. Baseline susceptibility to all US-approved influenza antivirals remained largely unchanged compared to previous seasons. All swine-origin viruses detected in the US had adamantane resistance-conferring marker, M2-S31N, but remained susceptible to other approved antivirals. Monitoring antiviral susceptibility has substantially improved with increased sequencing capacities and bioinformatic support at public health laboratories. Information gained through influenza surveillance has been used to guide recommendations on antiviral use.

**IMPORTANCE:**

Circulation of influenza viruses with reduced susceptibility to antivirals can diminish the usefulness of medications prescribed for influenza. This study informs on the prevalence of drug-resistant influenza viruses in the US during the high severity season of 2024–25. It provides information on susceptibility profile to all approved antiviral medications and on replicative fitness of representative drug-resistant viruses. Most drug-resistant viruses were collected from patients who were not exposed to antivirals indicating their ability to transmit from human to human. Whole-genome sequence (WGS)-based analysis is the cornerstone for surveillance, and numerous laboratories have been utilizing this approach. However, CDC laboratory is the only laboratory in the US conducting phenotypic testing of circulating viruses needed to confirm the outcomes of sequence-based analysis and to identify new molecular markers of resistance. Data gathered through virologic surveillance give much-needed information on drug susceptibility of influenza viruses which are used to guide recommendations on antiviral use.

## INTRODUCTION

The 2024–25 influenza season in the United States (US) was characterized as the first high severity season for all ages since 2017–18 based on influenza-like illness visits to outpatient clinics, influenza-related hospitalizations, and percentage of deaths resulting from influenza (https://www.cdc.gov/flu/php/surveillance/index.html and https://www.cdc.gov/flu/php/surveillance/past-seasons.html). Two influenza A subtypes A(H1N1)pdm09 and A(H3N2) circulated at approximately equal proportions during this season (https://www.cdc.gov/flu/whats-new/2025-2026-influenza-activity.html). While seasonal influenza vaccination remains the primary tool to control influenza, antiviral treatment can reduce the duration of symptoms, prevent some influenza-related complications, and reduce household transmission (1, 2). During high severity influenza seasons, the usage of antiviral medications might increase, which may lead to the emergence of drug-resistant viruses (3, 4).

Adamantanes (Matrix-2 (M2) channel blockers) are not recommended to treat seasonal influenza virus infections as nearly all circulating viruses are resistant to this class of antivirals (5, 6). Four other US Food and Drug Administration approved influenza antivirals are neuraminidase (NA) inhibitors oseltamivir, zanamivir, and peramivir; and baloxavir, the first-in-class inhibitor of the polymerase acidic (PA) cap-dependent endonuclease (aka PA inhibitor). Zanamivir is delivered by inhalation and peramivir by intravenous route, whereas oseltamivir and baloxavir are oral medications (https://www.cdc.gov/flu/treatment/antiviral-drugs.html and https://www.cdc.gov/flu/hcp/antivirals/summary-clinicians.html). The latter drug was approved in 2018 and is prescribed as a single dose. In the US and other countries, oseltamivir remains the most prescribed antiviral for influenza (7, 8).

Influenza viruses can become less susceptible to antivirals by acquiring mutations that alter the drug’s target site (9). Such mutations can occur during virus replication in the presence of antivirals or spontaneously. Numerous amino acid substitutions in drug-targeted viral proteins have been implicated in reduced antiviral susceptibility. For NA inhibitors, mutations produce a type- and subtype-specific effect on drug susceptibility (https://cdn.who.int/media/docs/default-source/influenza/laboratory---network/quality-assurance/human-nai-marker-table.pdf). While some mutations affect binding of a single antiviral, others can affect binding of multiple NA inhibitors. There are no laboratory correlates of clinically relevant resistance to NA inhibitors, with one exception. The amino acid substitution H275Y in NA of N1 subtype viruses is considered an established molecular marker of oseltamivir resistance (10). This mutation also confers resistance to peramivir but does not affect susceptibility to zanamivir. Oseltamivir-resistant A(H1N1) viruses with H275Y circulated widely during 2007–2009. “Permissive” NA mutations have previously been associated with an improved viral fitness of these A(H1N1) viruses with H275Y, contributing to their rapid global spread (11). These viruses ceased to circulate after the 2009 A(H1N1)pdm09 pandemic. In the years following its introduction and establishment as a new seasonal strain, community clusters of A(H1N1)pdm09 viruses with H275Y have also been reported in different countries (6, 12–14). Some NA substitutions, such as NA-R257K, V234M, T289M, or N369K, were previously associated with restoring NA activity of A(H1N1)pdm09 viruses with H275Y (12, 14–16).

For baloxavir, I38T in the PA subunit of the viral RNA polymerase is considered the principal molecular marker of reduced susceptibility although numerous other PA substitutions have also been reported (17, 18, https://cdn.who.int/media/docs/default-source/influenza/laboratory---network/quality-assurance/antiviral-susceptibility-influenza/pa-marker-who-table_28-11-2025_updated.pdf). Similar to NA inhibitors, there are no established correlates of clinically relevant baloxavir resistance, and for the purpose of virologic surveillance, test results are interpreted using a provisional cutoff (>3-fold rise in EC_50_) (19). Cell culture-based infectivity assays are used to determine susceptibility of viruses to baloxavir, but not to NA inhibitors as this approach has proven unreliable for this class of antivirals (8). Instead, viruses are tested using the NA inhibition (NI) assay, which determines the drug concentration needed to inhibit the NA enzyme activity.

The aim of antiviral susceptibility surveillance is to detect changes in viruses that may reduce antiviral effects of therapeutics. Rather than detecting drug-emergent resistance, antiviral susceptibility surveillance focuses on detecting the spread of drug-resistant viruses in communities. Therefore, specimens submitted for virologic surveillance are typically collected before initiation of antiviral treatment or prophylaxis.

In the US, antiviral susceptibility of influenza viruses is monitored by CDC in collaboration with the Association of Public Health Laboratories (APHL) as part of virologic surveillance (6, 20). This surveillance is conducted year-round; however, efforts are heightened from October to mid-May. To boost antiviral surveillance at the state-level, additional specimens are sent by state public health laboratories to the designated laboratory at the National Influenza Reference Center (NIRC), New York State Department of Health (Wadsworth Center), in Albany, New York (NY). Furthermore, two other NIRCs at the California (CA) Department of Public Health Viral and Rickettsial Disease Laboratory and Wisconsin (WI) State Laboratory of Hygiene and six Influenza Sequencing Centers (ISCs) also participate in state-level virologic surveillance on additional samples collected in their respective states. Specimens that are received undergo complete-codon, whole-genome sequencing, and viral genome sequences are then shared with CDC for comprehensive analysis. CDC performs testing of representative seasonal and swine-origin (variant) viruses with antivirals using established laboratory assays. In addition to US specimens, CDC conducts testing of influenza viruses from other countries participating in the Global Influenza Surveillance and Response System (GISRS).

In this study, we provide the outcomes of antiviral susceptibility surveillance conducted in the US during the high severity influenza season of 2024–25 and characterized viruses with reduced susceptibility to approved influenza antivirals. We also included results for swine-origin variant viruses collected from humans during 2024 and 2025.

## MATERIALS AND METHODS

### Influenza viruses collected during 2024–25

Influenza positive specimens were analyzed by the CDC-led virologic surveillance network, which encompasses three parts: US national, US supplemental in-state, and GISRS National Influenza Centers outside of the US (6).

For US national surveillance, submission of 6 A(H3N2), 4 A(H1N1)pdm09, and 4 B/Victoria lineage positive clinical specimens by state public health laboratories (PHLs) every 2 weeks was carried out according to the seasonal Right Size CDC/APHL guidance (https://www.aphl.org/aboutAPHL/publications/Documents/ID-Influenza-Right-Size-Roadmap-Edition2.pdf). A total of 5,487 specimens (2,148 A(H1N1)pdm09, 2,411 A(H3N2), and 928 B/Victoria) were received by three NIRCs (Table S1). Whole-genome sequencing (WGS) of nearly all (99%) viruses and virus isolation of 3,352 specimens (61% of received) were carried out by three NIRCs, and WGS data and virus isolates were transferred to CDC for further analysis and testing.

For US supplemental surveillance at the state-level, additional 5,416 viruses (2,624 A(H1N1)pdm09, 2,281 A(H3N2), and 511 B/Victoria) collected in 18 states were received for sequence-based antiviral susceptibility monitoring (Table S1). For this arm of surveillance, a designated NIRC (NY) laboratory used either pyrosequencing or WGS to detect drug-resistant viruses, while other laboratories (NIRCs at CA and WI and ISCs at Colorado, Florida, Hawaii, Massachusetts, Minnesota, and Texas) used WGS only.

CDC laboratory also received 2,317 influenza viruses (952 A(H1N1)pdm09, 675 A(H3N2), and 690 B/Victoria) from 51 other countries; and 90% and 61% of them were sequenced and propagated in cell culture, respectively (Table S1).

Swine-origin (variant) viruses were submitted by state PHLs to CDC. Handling and testing of these viruses were done in Biosafety Level 2 enhanced laboratories.

### Cell cultures and virus isolation

MDCK (ATCC), MDCK-SIAT1 (Millipore Sigma, 21), and humanized MDCK (hCK, kindly provided by Dr. Kawaoka, 22) cells were used in this study. All A(H3N2) and most of A(H1N1)pdm09 viruses were isolated in MDCK-SIAT1 cells; while type B, some of A(H1N1)pdm09, and swine-origin (variant) viruses were propagated in MDCK cells.

### Whole-genome sequencing

Codon-complete influenza genome was amplified using a Uni/Inf primer set as previously described (23). Index paired-end libraries were prepared using Illumina DNA Prep library preparation kits and sequenced using either MiSeq or NextSeq 550 (Illumina). The next-generation sequencing and assembly pipeline, IRMA, was used for alignment, assembly, and consensus determination (24). Apart from IRMA’s quality control metrics, sequences were also required to meet additional passing criteria as described previously (6). Sequences were deposited into influenza sequence databases, Global Initiative on Sharing All Influenza Data (GISAID; EPI_SET_260401bk, 25), and National Center for Biotechnology Information (NCBI).

### Sequence and phylogenetic analysis

Generated NA and PA sequences were screened for the presence of molecular markers, which are amino acid substitutions known or suspected to confer reduced antiviral susceptibility (https://cdn.who.int/media/docs/default-source/influenza/laboratory---network/quality-assurance/human-nai-marker-table.pdf and https://cdn.who.int/media/docs/default-source/influenza/laboratory---network/quality-assurance/antiviral-susceptibility-influenza/pa-marker-who-table_28-11-2025_updated.pdf). PA sequences were flagged for known or new substitutions at 25 amino acid residues, most within the N-terminal cap-dependent (CEN) domain (Table S2a). The substitutions at any of these residues were categorized into the four groups: primary, secondary, tertiary, and new substitutions (Table S2b). Additionally, sequence analysis was expanded by including complete NA sequences of A(H1N1)pdm09 viruses submitted to GISAID by other laboratories (accessed on 19 March 2026; EPI_SET_260319dz), which were analyzed for the presence of H275Y and other NA mutations (e.g., R257K). Hemagglutinin (HA) and NA clade/subclade designations were assigned by Nextclade (26, 27).

### Antivirals

NA inhibitors, oseltamivir carboxylate (oseltamivir), zanamivir, peramivir, and laninamivir (Biosynth) and PA inhibitor baloxavir acid (baloxavir, active metabolite of prodrug, MedChem Express) were used for antiviral susceptibility assays.

### Antiviral susceptibility assays

Virus susceptibility to NA inhibitors was assessed using the fluorescence-based NI assay (28), while susceptibility to baloxavir was evaluated using the cell culture-based Influenza Replication Inhibition NA-based assay (IRINA, 29). Representative seasonal viruses from each type/subtype were selected for testing based on genetic clades, geography, and date of collection. When assessing NA inhibitor baselines (median IC_50_), we not only excluded IC_50_s of viruses with known NA markers of reduced or highly reduced inhibition (RI or HRI) but also those that are known to elevate IC_50_ by less than 10-fold (e.g., S247N).

Fold change in NA inhibitor IC_50_ or baloxavir EC_50_ of a test virus was calculated relative to a subtype- or lineage-specific median value generated by testing representative viruses and, if available, sequence-matched control virus. For swine-origin (variant) viruses, subtype-specific medians were determined in our previous study (30). Interpretation of testing outcomes was done according to the arbitrary criteria adopted by surveillance laboratories (31, 32). Fold change in NA inhibitor IC_50_ was interpreted as follows: normal inhibition (type A < 10-fold; type B < 5-fold); RI (type A 10- to 100-fold; type B 5- to 50-fold), or HRI (type A > 100-fold; type B > 50-fold). Fold change in baloxavir EC_50_: normal susceptibility (≤3-fold) and reduced susceptibility (>3-fold) for both type A and B viruses (19, 31).

### *In vitro* replicative fitness

The MDCK, MDCK-SIAT1, and hCK cell monolayers in six-well plates were inoculated at a multiplicity of infection of ~0.0001 in duplicate. Following virus adsorption for 30 min at 37°C, virus growth media supplemented with 2 µg/mL TPCK-trypsin was added, and incubation was continued at 37°C in 5% CO_2_ for 72 h. Cell supernatants were collected at indicated time points to determine viral titers (TCID_50_/mL). Average virus titers at each time point from duplicate experiments were plotted. The control wild-type viruses used for comparison had only one or two amino acid differences in the HA or NA compared to PA-I38-substituted viruses.

## RESULTS

### Detection of oseltamivir-resistant A(H1N1)pdm09 viruses with H275Y

**Fig 1:**
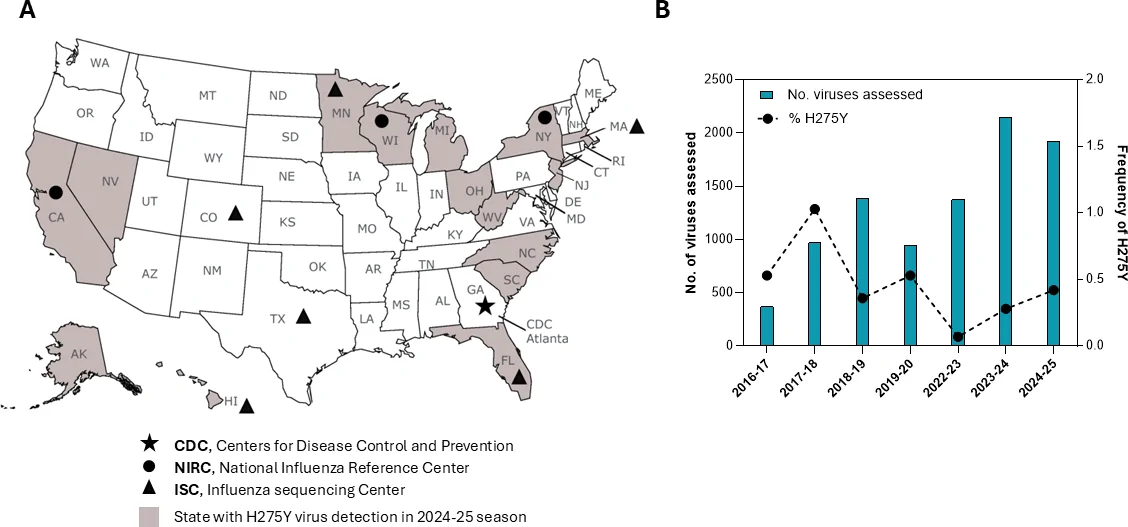
Detection of influenza A(H1N1)pdm09 virus with H275Y in the US. (**A**) 15 US states with H275Y virus detection in the 2024–25 season through national and supplemental in-state surveillance are shown with light gray shading. Through national surveillance, eight H275Y viruses were detected in seven states (two in Nevada and one each in Hawaii, North Carolina, New Jersey, Ohio, South Dakota, West Virginia). Through supplemental surveillance, 16 H275Y viruses were detected in 10 states (3 in Florida, 2 in each of Hawaii, Massachusetts, Minnesota, and Wisconsin, and 1 each in Alaska, California, Michigan, North Carolina, and New York). Location of CDC (Georgia), three NIRCs (California, New York, and Wisconsin), and six ISCs (Colorado, Florida, Hawaii, Massachusetts, Minnesota, and Texas) in the US state is shown. The geographical US map was generated using Microsoft Power BI Desktop software employing its “shape map function.” Additionally, the Microsoft Power BI Desktop generated map was enhanced for visualization using Adobe Illustrator. (**B**) Frequency of H275Y detection in the US via national surveillance during the 2016–25 seasons (excluding 2020–21 and 2021–22 seasons with very low number of influenza viruses in circulation due to COVID-19 pandemic). All seasons span from 1 October to 30 September of the following year.

The US national surveillance detected H275Y in 8 of 2,129 A(H1N1)pdm09 viruses (0.38%) collected in 7 states (Fig. 1A; Table 1). Notably, in most instances, H275Y viruses were collected from patients who were not exposed to oseltamivir (Table 1). In previous seasons, the frequency of H275Y detection varied from 0.07% to 1.03%, with the highest seen in the high severity season of 2017–18 (Fig. 1B). During the 2024–25 season, 16 of 2,379 A(H1N1)pdm09 viruses analyzed (0.67%) through supplemental in-state surveillance had H275Y and were collected in 10 states (Fig. 1A; Table 1). Of 886 A(H1N1)pdm09 viruses collected in 51 other countries, H275Y was detected in 5, 2 of which were collected in the United Arab Emirates and 1 each in Argentina, Belgium, and Paraguay (Table 1).

**TABLE 1:** Summary of influenza A and B viruses with H275Y or other NA substitutions associated to confer reduced or highly reduced inhibition by NA inhibitors, CDC-led surveillance 2024–25^*a*^

Surveillance arm	Virus name	Type/subtype or lineage	Date of collection (MM/DD/Yr)	Patient’s location (county/state/country)^*b*^	Patient age/sex	NA substitution^*c*^		NA sequence GISAID acc. no.^*d*^	NAI exposure
						Clinical sp.	Virus isolate		
Viruses with H275Y									
US National	A/Hawaii/12/2025	A(H1N1)pdm09	3/19/2025	Honolulu, Honolulu Co.	13/F	H275Y	Not available	EPI4276205	No
US National	A/Nevada/01/2025	A(H1N1)pdm09	1/1/2025	Gardnerville, Douglas Co.	4/Unk	H275Y	H275Y	EPI3952295	No
US National	A/Nevada/251/2024	A(H1N1)pdm09	12/26/2024	Fallon, Churchill Co.	26/F	H275Y	H275Y	EPI3951757	No
US National	A/New Jersey/27/2025	A(H1N1)pdm09	2/17/2025	Glassboro, Gloucester Co.	21/F	H275Y	H275Y	EPI4132067	No
US National	A/North Carolina/30/2025	A(H1N1)pdm09	2/6/2025	Forsyth Co.	65/F	H275Y	H275Y	EPI4221781	Yes, osel
US National	A/Ohio/127/2025	A(H1N1)pdm09	3/19/2025	Cincinnati, Hamilton Co.	75/M	H275Y	VNR	EPI5348026	Yes, osel
US National	A/South Dakota/08/2025	A(H1N1)pdm09	1/6/2025	Faulkton	12/M	H275Y	H275Y	EPI3951849	No
US National	A/West Virginia/70/2024	A(H1N1)pdm09	12/23/2024	Whitman, Boone Co.	0/M	H275Y/H (Y 18%)	H275Y/H (Y 12%)	EPI3952251	No
US Supplemental (CA NIRC)	A/California/NIRC-IS-1234/2025	A(H1N1)pdm09	5/19/2025	California	1/F	H275Y	–	EPI4475468	Unk
US Supplemental (NY NIRC)	-	A(H1N1)pdm09	02/06/25	Nenana, Alaska	81/M	H275Y	–	PYRO	Unk
US Supplemental (NY NIRC)	-	A(H1N1)pdm09	2/13/2025	Onondaga, New York	54/F	H275Y	–	PYRO	Unk
US Supplemental (NY NIRC)	A/Michigan/193/2025	A(H1N1)pdm09	3/26/2025	Wayne Co.	70/M	H275Y	–	Not available	Unk
US Supplemental (NY NIRC)	-	A(H1N1)pdm09	03/30/25	Catawba Co., North Carolina	19/M	H275Y	H275Y	PYRO	Unk
US Supplemental (WI NIRC)	A/Wisconsin/NIRC-IS-1139/2025	A(H1N1)pdm09	2/22/2025	Wisconsin	Unk	H275Y	–	EPI4150364	Unk
US Supplemental (WI NIRC)	A/Wisconsin/NIRC-IS-1224/2025	A(H1N1)pdm09	3/21/2025	Wisconsin	Unk	H275Y	–	EPI4279356	Unk
US Supplemental (FL ISC)	A/Florida/ISC-1375/2024	A(H1N1)pdm09	12/7/2024	Hollywood, Broward Co.	3/F	H275Y	–	EPI3940930	Unk
US Supplemental (FL ISC)	A/Florida/ISC-1162/2025	A(H1N1)pdm09	2/7/2025	Pensacola, Escambia Co.	55/F	H275Y	–	EPI4050158	Unk
US Supplemental (FL ISC)	A/Florida/ISC-1211/2025	A(H1N1)pdm09	2/23/2025	Stuart, Martin Co.	71/M	H275Y	–	EPI4195737	Unk
US Supplemental (HI ISC)	A/Hawaii/ISC-1187/2025	A(H1N1)pdm09	4/11/2025	Hawaii	80/F	H275Y	–	EPI4328680	Unk
US Supplemental (HI ISC)	A/Hawaii/ISC-1211/2025	A(H1N1)pdm09	5/20/2025	Hawaii	7/M	H275Y	–	EPI4446024	Unk
US Supplemental (MA ISC)	A/Massachusetts/ISC-1486/2025	A(H1N1)pdm09	2/7/2025	Massachusetts	64/F	H275Y	–	EPI4049395	Unk
US Supplemental (MA ISC)	A/Massachusetts/ISC-1781/2025	A(H1N1)pdm09	3/20/2025	Massachusetts	59/F	H275Y + S247N	–	EPI4406177	Unk
US Supplemental (MN ISC)	A/Minnesota/ISC-1170/2025	A(H1N1)pdm09	2/17/2025	Minnesota	1/F	H275Y	–	Not available	Unk
US Supplemental (MN ISC)	A/Minnesota/ISC-1345/2025	A(H1N1)pdm09	3/17/2025	Minnesota	65/M	H275Y	–	EPI4278831	Unk
CDC/other countries	A/Abu Dhabi/9379/2024	A(H1N1)pdm09	10/28/2024	United Arab Emirates	7/M	H275Y	H275Y	EPI3760272	Unk
CDC/other countries	A/Abu Dhabi/0866/2024	A(H1N1)pdm09	11/11/2024	United Arab Emirates	4/M	H275Y	H275Y	EPI3760436	Unk
CDC/other countries	A/Argentina/1418/2025	A(H1N1)pdm09	6/21/2025	Argentina	77/M	H275Y	H275Y	EPI4632179	Unk
CDC/other countries	A/Belgium/0033/2025	A(H1N1)pdm09	2/4/2025	Belgium	33/F	H275Y + S247N	H275Y + S247N	EPI4617482	Unk
CDC/other countries	A/Paraguay/9601/2025	A(H1N1)pdm09	6/6/2025	Paraguay	39/F	H275Y	H275Y	EPI4738817	Unk
Viruses with other NA substitutions									
US National	A/Pennsylvania/30/2025	A(H1N1)pdm09	2/23/2025	Hiladelphia, Philadelphia Co.	36/F	S247R	S247R	EPI4188342	Unk
US National	A/Minnesota/104/2025	A(H1N1)pdm09	9/8/2025	Edina, Hennepin Co.	Unk	I223V + S247N	I223V + S247N	EPI4779580	Unk
US National	A/California/189/2024	A(H1N1)pdm09	10/16/2024	San Jose, Santa Clara Co.	55/F	I223V, S247N	I223V, S247N	EPI4248958	Unk
US National	A/New York/28/2025	A(H1N1)pdm09	02/25/2025	Brooklyn, Kings Co.	56/F	I223M	Not available	EPI4080249	Unk
US National	A/New York/17/2025^*e*^	A(H3N2)	1/13/2025	Commack, Suffolk Co.	4/F	E119V	E119V	EPI3956761	Yes, osel
US National	A/Indiana/60/2024^*e*^	A(H3N2)	12/26/2024	Avon	11/F	E119V/E (V 19%), R292K/R (K 75%)	E119V/E (V 61%), R292K/R (K 13%)	EPI3953643	Yes, osel
US National	B/Minnesota/12/2025	B/Victoria	3/11/2025	Saint Peter	Unk	A245D	A245D	EPI4302163	Unk
US Supplemental (CA NIRC)	A/California/NIRC-IS-1151/2025	A(H1N1)pdm09	1/16/2025	California	Unk	S247G	–	EPI4406717	Unk
US Supplemental (CA NIRC)	A/California/NIRC-IS-1171/2025	A(H1N1)pdm09	1/20/2025	California	Unk	S247G	–	EPI4406764	Unk
US Supplemental (NY NIRC)	B/New York/NIRC-IS-1005/2025	B/Victoria	3/18/2025	New York	Unk	D197E	–	EPI4406383	Unk
US Supplemental (WI NIRC)	A/Wisconsin/NIRC-IS-1046/2025	A(H1N1)pdm09	1/12/2025	Wisconsin	Unk	S247G/S	–	EPI3938823	Unk
US Supplemental (MA ISC)	A/Massachusetts/ISC-1096/2024	A(H3N2)	11/3/2024	Massachusetts	6 M	R292K/R	–	EPI3675203	Unk
CDC/other countries	A/British Columbia/Rv04868/2024	A(H1N1)pdm09	10/14/2024	Canada	Unk	Unk	I223K	EPI3794746	Unk
CDC/other countries	B/Ghana/6028/2025	B/Victoria	8/1/2025	Ghana	34/F	H273Y	VNR	EPI4758658	Unk

^*a*^ GISAID, Global Initiative on Sharing All Influenza Data; NA, neuraminidase; NAI, neuraminidase inhibitor; Osel, oseltamivir; PYRO, pyrosequencing; Unk, unknown; VNR, virus not recovered; “–” not applicable.

^*b*^ If available, patient’s city or county information is provided, otherwise US state. For viruses from other countries, name of the country is provided.

^*c*^ Substitution is shown using type- or subtype-specific NA amino acid numbering system.

^*d*^ NA sequence number of the original clinical specimen.

^*e*^ These viruses were collected from immunocompromised patients.

### Phylogeny of H275Y viruses and NA permissive mutations

To further characterize the H275Y viruses, we performed HA and NA phylogenetic analysis. H275Y viruses detected in the US through CDC surveillance belonged to several distinct HA and NA subclades, with the most common HA-NA combination of HA 5a.2a.1_D.3.1 and NA subclade D.1 (Table S3). The 3 H275Y viruses detected in Florida belonged to different HA and NA subclades, while 2 of the 3 that were detected in Hawaii belonged to the most common HA-NA combination. The 2 H275Y viruses detected in Nevada shared identical genomes and belonged to a less common HA-NA combination, HA 5a.2a_C.1.9, and NA subclade D (Table S3). The latter viruses were detected 1 week apart from patients who had no exposure to oseltamivir and who resided in neighboring counties (Table 1). These data indicate potential local transmission of oseltamivir-resistant viruses in Nevada albeit at a low frequency (2 of 91 sequences analyzed).

We expanded the analysis of H275Y viruses by including NA sequences deposited by other laboratories in GISAID EpiFlu database. Among 34 additional H275Y viruses from the US, only three belonged to the same group as the H275Y viruses from Nevada, while most of the others belonged to HA clade 5a.2a.1_D.3.1, with either NA subclade D.1 or D.2 (Tables S3 and S4). Nearly all viruses with H275Y that were collected in Australia (52/57), Canada (16/18), and Chile (12/12) belonged to the common HA and NA subclades seen in the US (Table S5). Most H275Y viruses from the United Kingdom, Japan, and Spain also belonged to NA subclade D.2, but a different HA clade and subclade 5a.2a_C.1.9.3. In China, most (70 of 81) H275Y viruses were also from this HA clade, but the subclade was different, 5a.2a_C.1.9, combined with NA subclade D (Table S5). Interestingly, most (84%, 69/81) of H275Y viruses from China had the permissive NA mutation R257K, known to improve virus fitness (Table S5, 16). This mutation was also present in nearly all H275Y viruses collected in China and Japan in the months preceding the 2024–25 season (https://nextstrain.org/seasonal-flu/h1n1pdm/na/3y?c=gt-NA_257,275&f_country=China,Japan&gmax=1418&gmin=9&p=full). In the 2024–25 season, H275Y viruses with R257K mutation were seen sporadically in several other countries (data not shown), including a single case in the US (Table S3). This permissive mutation has been introduced multiple times into viruses circulating globally, with a peak of >20% in June 2024. However, unlike the permissive NA mutation N369K, which has been fixed since 2013, R257K seems to have disappeared from circulation by the end of 2025 (33, https://nextstrain.org/seasonal-flu/h1n1pdm/na/6y?c=gt-NA_257&gmax=1418&gmin=9).

### Detection of other NA molecular markers

Through the CDC surveillance, 8 A(H1N1)pdm09, 3 A(H3N2), and 3 B/Victoria viruses were detected to have previously reported or suspected NA molecular marker of interest (Table 1). All sequence-flagged viruses, except 2 (1 A(H1N1)pdm09 with I223K from Canada and 1 B/Victoria with H273Y from Ghana) were collected in the US. In A(H1N1)pdm09, these NA markers were I223K (Canada), I223M, S247G, S247R, or I223V + S247N. By itself, S247N confers <10-fold elevation in IC_50_ for oseltamivir and, therefore, is not a reportable NA marker. However, it can potentiate the effect of other NA substitutions (e.g., H275Y, 34). In this study, we detected two H275Y viruses with S247N (Table 1). In recent years, there were multiple introductions of S247N into different NA subclades (35, https://nextstrain.org/seasonal-flu/h1n1pdm/na/6y?c=gt-NA_247&gmax=1418&gmin=9&p=full). During 2024–25, the frequency of S247N remained low (1.8%; 79/4324) in the US observed via CDC surveillance; but it increased in the months preceding the winter season of 2025–26; a similar trend was seen in other regions, including several countries in Europe (36).

Among A(H3N2) viruses, 1 had E119V, while another had a mixed population at 119 (E and V) and 292 (R and K) (Table 1). In both instances, specimens were collected from immuno-compromised children following oseltamivir treatment. The third A(H3N2) virus also had a mixture of R and K at 292. One B/Victoria virus had H273Y (Ghana), 1 had D197E, and another one had A245D; the latter is a new substitution at a residue previously associated with RI by NA inhibitors (Table 1, https://cdn.who.int/media/docs/default-source/influenza/laboratory---network/quality-assurance/human-nai-marker-table.pdf).

### NA inhibitor susceptibility by NI assay

**Fig 2:**
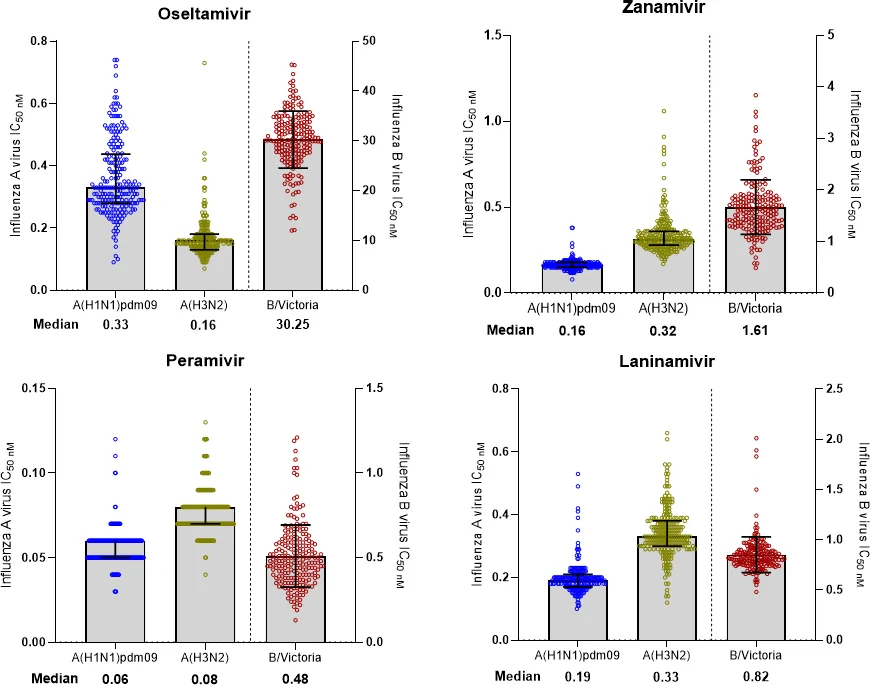
Assessment of baseline NA inhibitor susceptibility of seasonal influenza viruses using NI assay, 2024–25 season. Representative influenza viruses (*n* = 657; 208 A(H1N1)pdm09, 246 A(H3N2), and 203 B/Victoria) lacking any NA substitutions of concern were tested in presence of oseltamivir, zanamivir, peramivir, and laninamivir to determine the type- or subtype-specific median IC_50_. In a scatter dot plot, median (gray bar; also shown on x-axis as number) and interquartile range are shown. For all four graphs, IC_50_s for both A subtype viruses were plotted using left *y*-axis, while IC_50_s for B/Victoria viruses were plotted using right *y*-axis.

To determine the 2024–25 seasonal susceptibility baseline (i.e., subtype/lineage-specific median IC_50_), a subset of viruses from the US and other countries was tested with NA inhibitors (Fig. 2). Overall, oseltamivir IC_50_ medians for type B/Victoria lineage viruses were >100-fold higher than type A viruses, which is consistent with our previous reports (6, 28, 37). Despite ongoing virus evolution, the 4 NA inhibitors continue to effectively inhibit NA enzyme activity of influenza A and B viruses with the baseline remaining largely unchanged from season to season (Fig. S1).

Available isolates of sequence-flagged viruses with H275Y and other NA substitutions were tested in NI assay and their IC_50_ were compared to type/subtype-specific medians (Table S6). A(H1N1)pdm09 viruses with H275Y displayed HRI by oseltamivir and peramivir, while the virus with H275Y + S247N showed an even greater reduction (Table S6). A(H1N1)pdm09 with I223K or I223V + S247N displayed RI by oseltamivir. A(H1N1)pdm09 with S247R conferred RI by oseltamivir and zanamivir and HRI by peramivir and laninamivir. One A(H3N2) virus with E119V displayed HRI by oseltamivir. Mixed viral populations in the second A(H3N2) virus were separated by a limiting dilution procedure. Viruses with E119V or R292K displayed HRI by oseltamivir; R292K also conferred RI by the other 3 NA inhibitors (Table S6). The observed IC_50_ fold-changes and their interpretation were consistent with previous reports (Table S6, https://cdn.who.int/media/docs/default-source/influenza/laboratory---network/quality-assurance/human-nai-marker-table.pdf). The newly identified A245D in B/Victoria virus conferred a 5- to 6-fold RI by zanamivir and peramivir.

### Sequence-based assessment of baloxavir susceptibility

To assess susceptibility to baloxavir, PA sequences of viruses from the US (*n* = 9,188) and 51 other countries (*n* = 1,820) were generated and analyzed according to the “augmented” approach, which our laboratory recently implemented (6, Table S2a). Substitutions at 25 residues within the PA were divided into four categories: (i) primary—reduced susceptibility, (ii) secondary—suspected reduced susceptibility, (iii) tertiary—normal susceptibility, and (iv) new substitutions with unknown effects (Table S2b).

In the primary category, I38T was detected in 1 A(H1N1)pdm09 and 1 A(H3N2) virus. In both instances, patients were from the US and had no exposure to baloxavir (Table 2). Prior to the 2024–25 season, the only time I38T was detected through US national surveillance was a single case in 2023, which was linked to recent travel from Japan (6). Another primary category substitution, I38M, was detected in 2 A(H3N2) viruses, 1 each from the US and Maldives. In the secondary category, K34R (*n* = 1), A37T (*n* = 1), and D27G (*n* = 4) were found in type A viruses from the US only. In the same category, E199D was detected in 3 type A viruses (1 each from the US, Chile, and South Africa) and E199G in 1 virus from Argentina (Table 2). In the tertiary category, in which effect on susceptibility is not expected, substitutions at residues 20, 24, 28, 38, 84, 120, or 122 were identified. Lastly, mutations with an unknown effect were E18K, Y24H, D27E, D27N, D27Y, L28R, P28S, A36T, and L42S (Table S7).

**TABLE 2:** Baloxavir susceptibility assessment of influenza A viruses with primary or secondary category PA substitutions using IRINA, CDC-led surveillance 2024–25^*a*^

PA substitution	Virus name	Type/subtype	Date of collection (MM/DD/YY)	Patient age/sex	Baloxavir exposure	Baloxavir EC_50_ nM^*b*^ Mean ± SD	Baloxavir EC_50_ fold change^*c*^		PA sequence GISAID acc. no.^*d*^
							vs control	vs median	
Primary									
I38T	A/Lousiana/30/2025	A(H1N1)pdm09	3/24/25	29/F	No	67.26 ± 22.43	91	84	EPI4301478
I38T	A/North Dakota/18/2025	A(H3N2)	1/25/25	85/M	No	105.67 ± 27.30	92	149	EPI4045029
I38M	A/Massachusetts/ISC-1099/2024	A(H3N2)	11/5/24	8/M	Unknown	10.26 ± 1.44	13	14	EPI3675224
I38M	A/Maldives/2410/2024	A(H3N2)	12/23/24	29/F	Unknown	13.89 ± 0.45	17	20	EPI3956070
Secondary									
K34R	A/Massachusetts/96/2025	A(H1N1)pdm09	7/5/25	82/F	Unknown	7.26 ± 0.39	7	9	EPI4645359
A37T	A/Iowa/19/2025	A(H3N2)	1/30/25	11/M	No	4.00 ± 0.82	5	6	EPI4008975
D27G	A/Massachusetts/28/2025	A(H1N1)pdm09	2/10/25	9/F	Unknown	2.02 ± 0.29	4	2.5	EPI4132080
D27G	A/Utah/31/2025	A(H1N1)pdm09	3/10/25	70/M	Unknown	–	–	–	EPI4248447
D27G	A/Massachusetts/ISC-1537/2025	A(H1N1)pdm09	1/24/25	47/M	Unknown	–	–	–	EPI4048652
D27G	A/New York/NIRC-IS-1376/2025	A(H1N1)pdm09	3/7/25	Unknown	Unknown	–	–	–	EPI4475481
E199D	A/Puertomontt/58949/2025^*e*^	A(H1N1)pdm09	7/14/25	7/F	Unknown	1.03 ± 0.28	1	1	EPI4719468
E199D	A/Colorado/104/2025	A(H3N2)	8/20/25	4/F	Unknown	0.72 ± 0.21	1	1	EPI4738460
E199D	A/South Africa/7928/2025	A(H3N2)	8/13/25	55/M	Unknown	–	–	–	EPI4822733
E199G	A/Argentina/5273/2025	A(H1N1)pdm09	5/8/25	10/F	Unknown	2.04 ± 0.73	2	2.6	EPI4758367

^*a*^ EC_50_, 50% effective concentration; IRINA, Influenza Replication Inhibition Neuraminidase-based Assay; PA, Polymerase acidic subunit; SD, standard deviation; “–” virus not recovered or available to test in IRINA.

^*b*^ Baloxavir susceptibility of isolates of sequence-flagged viruses was assessed using a cell culture-based Influenza Replication Inhibition Neuraminidase-based Assay (IRINA). Viruses were tested in at least 2 independent tests.

^*c*^ Fold changes in EC_50_s were calculated vs PA sequence matched control virus or subtype-specific median EC_50_s generated by testing representative viruses circulated during 2024–25 season (see Table 3). Fold change was interpreted based on provisional cut-off used previously by surveillance laboratories, according to which >3-fold increase in EC_50_ of a test virus versus reference EC_50_ is defined as reduced susceptibility to baloxavir.

^*d*^ PA sequence number of the original clinical specimen.

^*e*^ This virus is from Chile.

**TABLE 3:** Baseline baloxavir susceptibility of seasonal influenza viruses assessed by IRINA, 2022–25 seasons^*a*^

Test viruses^*b*^	EC_50_ nM								
	2022–23			2023–24			2024–25		
	Mean ± SD	Range	Median	Mean ± SD	Range	Median	Mean ± SD	Range	Median
A(H1N1)pdm09	0.75 ± 0.31	0.24–1.69	0.71	0.88 ± 0.37	0.16–1.86	0.82	0.84 ± 0.31	0.34–1.76	0.80
A(H3N2)	0.74 ± 0.29	0.25–1.96	0.69	0.67 ± 0.26	0.15–1.64	0.63	0.76 ± 0.30	0.14–1.66	0.71
B/Victoria	4.69 ± 1.26	1.63–8.08	4.49	4.08 ± 1.33	1.20–8.74	3.87	3.44 ± 1.11	1.33–6.92	3.41
Reference viruses^*c*^	Mean ± SD	Range	Fold	Mean ± SD	Range	Fold	Mean ± SD	Range	Fold
A/IL/08/2018	2.08 ± 0.69	1.14–3.26	1.0	1.70 ± 0.44	0.91–3.03	1.0	1.77 ± 0.58	0.51–3.44	1.0
A/IL/37/2018, PA-I38L	17.15 ± 5.03	8.14–24.68	8.2	14.93 ± 3.24	7.20–21.63	8.8	16.39 ± 5.02	7.63–27.25	9.3
A/LA/50/2017	1.15 ± 0.34	0.54–2.24	1.0	1.00 ± 0.24	0.54–1.62	1.0	0.93 ± 0.25	0.51–1.76	1.0
A/LA/49/2017, PA-I38M	12.59 ± 3.44	6.97–24.92	10.9	11.94 ± 2.49	7.20–20.60	11.9	10.86 ± 2.73	6.36–17.63	11.7

^*a*^ EC_50_, 50% effective concentration; IL, Illinois; IRINA, Influenza Replication Inhibition Neuraminidase-based Assay; LA, Louisiana; SD, standard deviation.

^*b*^ A(H1N1)pdm09 (*n* = 283, 225, and 285), A(H3N2) (*n* = 185, 230, and 296), and B/Victoria lineage (*n* = 147, 244, and 236) viruses were tested 2022–23, 2023–24, and 2024–25, respectively, in the presence of baloxavir to determine the type- or subtype-specific baseline susceptibility. The test viruses did not contain any known PA substitutions of concern.

^*c*^ PA-I38L and PA-I38M viruses and their PA sequence-matched counterparts lacking these substitutions are from the CDC PA inhibitor susceptibility reference virus panel. Mean ± SD of EC_50_s and fold change conferred by mutant viruses were calculated based on results from at least 15 independent tests.

In B/Victoria lineage, as in previous seasons, no primary or secondary category substitutions were found. In the tertiary category, M34I, V121I, and G199R, as well as the new mutations D27N and I200V, were flagged for phenotypic testing (Table S7).

### Baloxavir susceptibility by the cell culture-based assay IRINA

Over 225 viruses of each type/subtype were tested using IRINA to assess the baseline susceptibility (median EC_50_) for the 2024–25 season. The testing outcomes were consistent with those from the previous two seasons (Table 3). As was seen before, the baseline for B/Victoria lineage remained ~5- to 6-fold higher compared to influenza A subtypes. Results for reference viruses were also consistent, with fold changes between PA mutant and wildtype remaining within <2-fold variation across the 3 seasons (Table 3).

The I38T substitution conferred 91- to 92-fold reduced susceptibility to baloxavir, while I38M conferred 13- to 17-fold (Table 2). The secondary category substitutions D27G, K34R, and A37T had a milder effect, 4- to 7-fold, while E199D and E199G had no effect (1- to 2-fold). Testing of available viruses with tertiary category and new substitutions confirmed their normal baloxavir susceptibility (Table S7).

Overall, CDC surveillance detected 10 viruses displaying reduced baloxavir susceptibility in the 2024–25 season; 9 of them from the US.

### Replicative fitness of viruses with I38T and I38M in PA polymerase subunit

**Fig 3:**
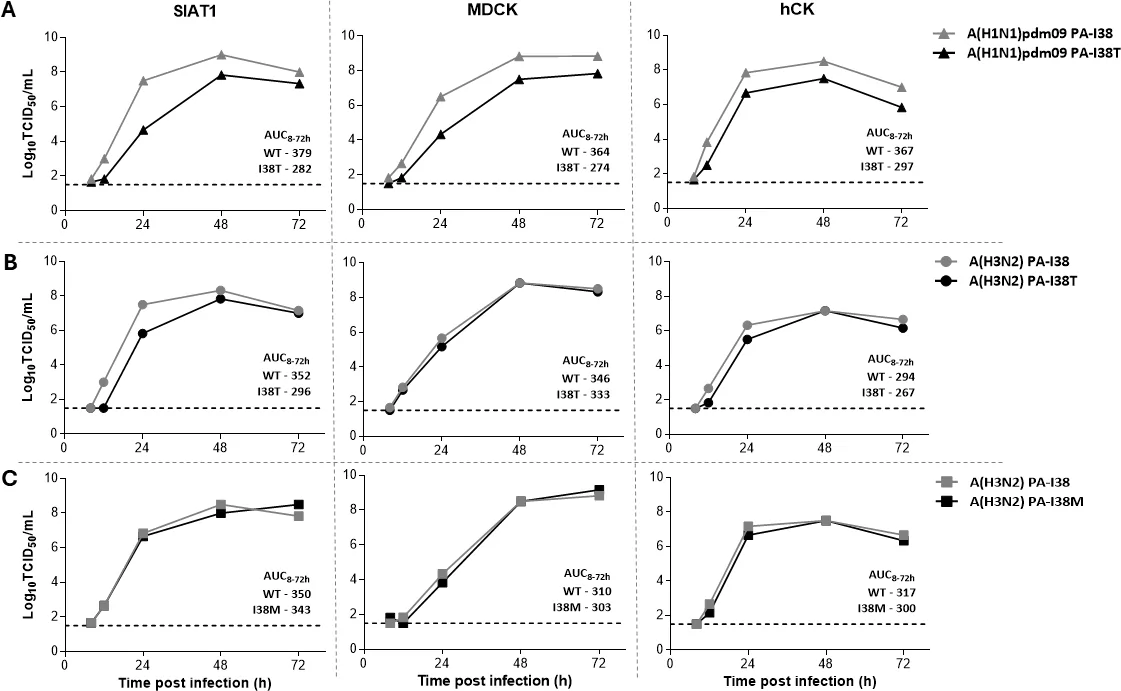
Effect of PA substitutions I38T or I38M on growth of type A viruses in MDCK-SIAT1 (SIAT1), MDCK, and hCK cells. Cell monolayers were inoculated at a multiplicity of infection of ~0.0001 with (A) A(H1N1)pdm09 PA-I38T and control, (B) A(H3N2) PA-I38T and control, and (C) A(H3N2) PA-I38M and control viruses. Supernatants were collected at 8, 12, 24, 48, and 72 h post-infection, and infectious virus titers were determined and expressed as log_10_TCID_50_/mL. The lower limit of virus detection is 1.50 log_10_ TCID_50_/mL (dashed line). The area under the curve for virus titers at 8–72 h post-infection (AUC_8–72_) was calculated using GraphPad Prism 10 and indicated in each graph for control (WT) and mutant viruses.

Information on replicative fitness of viruses displaying reduced baloxavir susceptibility remains sparse. Therefore, we assessed the *in vitro* growth kinetics of 3 clinical isolates with I38T or I38M detected during the 2024–25 season (Fig. 3).

Compared to the respective control virus, A(H1N1)pdm09 with I38T grew slower in all 3 cell lines with at least a 1 log_10_ TCID_50_/mL decrease in titers at several time points. Area under the curve of virus titers at 8–72 h (AUC_8–72h_) for I38T virus was 19.1%–25.6% lower than control virus (Fig. 3A). The A(H3N2) virus with the same mutation grew to the same titers as control virus in MDCK cells, but in SIAT1 and hCK, titers were lower by 0.83–1.67 log_10_ TCID_50_/mL at early time points (Fig. 3B). AUC_8–72h_ was 9.2%–15.9% lower for the I38T virus in SIAT1 and hCK cells, whereas it was only 3.8% in MDCK cells compared to control. On the other hand, A(H3N2) with I38M showed no apparent attenuation as its titers were comparable to control virus across 3 cell lines at all time points (Fig. 3C).

### Antiviral susceptibility of swine-origin (variant) viruses

A total of 9 variant influenza viruses from 3 subtypes, A(H1N1)v (*n* = 1), A(H1N2)v (*n* = 4), and A(H3N2)v (*n* = 4) were reported in 7 US states from May 2024 to October 2025 (https://gis.cdc.gov/grasp/fluview/Novel_Influenza.html). The analysis of available sequences (*n* = 8) revealed that all contained S31N in M2 protein, a marker of adamantane resistance (Table 4). One A(H1N1)v virus had V149I in NA, whose effect in this virus background on susceptibility to NA inhibitors is unknown. This change was flagged because another substitution at the same residue, V149A, was reported to confer ~8-fold RI by zanamivir in a clade 1 A(H5N1) virus (38). The virus with V149I (1-fold by all 4 NA inhibitors) and other available isolates were tested in NI assay and IRINA, and all were deemed susceptible to antivirals tested (Table 4).

**TABLE 4:** Antiviral susceptibility of swine-origin (variant) viruses collected in the US, May 2024 to October 2025^*a*^

Sub(type)	Variant virus	Collection date (MM/DD/YY)	NA inhibitor IC_50_ nM (fold vs median)				Baloxavir EC_50_ nM (fold vs median)	GISAID ID (EPI_ISL)
			Oseltamivir	Zanamivir	Peramivir	Laninamivir		
A(H1N1)v	2013–24 median (*n* = 15)^*b*^	–	0.43	0.21	0.07	0.22	0.75	–
	A/Ohio/251/2024^*c*^	8/7/24	0.54 ± 0.26 (1.3)	0.22 ± 0.02 (1.0)	0.07 ± 0.01 (1.0)	0.27 ± 0.07 (1.2)	1.48 ± 0.44 (2.0)	19339462
A(H1N2)v	2013–24 median (*n* = 14)^*b*^	–	0.20	0.55	0.16	0.92	0.98	–
	A/Pennsylvania/228/2024	6/17/24	0.24 ± 0.03 (1.2)	0.91 ± 0.08 (1.7)	0.21 ± 0.01 (1.3)	1.45 ± 0.27 (1.6)	0.81 ± 0.21 (0.8)	19226470
	A/Pennsylvania/242/2024	8/5/24	0.42; 0.34 (1.9)	1.08; 1.45 (2.3)	0.23; 0.35 (1.8)	1.37; 2.43 (2.1)	0.54 ± 0.15 (0.5)	19358668
	A/Vermont/75/2025	10/3/25	0.28; 0.25 (1.3)	0.73; 0.86 (1.4)	0.21; 0.18 (1.2)	1.02; 1.18 (1.2)	1.74 ± 0.32 (1.8)	20250635
A(H3N2)v	2013–24 median (*n* = 21)^*b*^	–	0.11	0.37	0.12	0.51	1.27	–
	A/Colorado/150/2024	7/9/24	0.15 ± 0.01 (1.4)	0.54 ± 0.10 (1.5)	0.17 ± 0.04 (1.4)	0.74 ± 0.20 (1.5)	2.09 ± 1.15 (1.6)	19328655
	A/Michigan/113/2024	7/24/24	0.15; 0.14 (1.3)	0.36; 0.57 (1.3)	0.11; 0.19 (1.3)	0.41; 0.76 (1.1)	2.44 ± 0.28 (1.9)	19339461
	A/Minnesota/100/2024	9/4/24	0.18 ± 0.01 (1.6)	0.57 ± 0.15 (1.5)	0.16 ± 0.04 (1.3)	0.76 ± 0.22 (1.5)	1.06 ± 0.42 (0.8)	19433719
	A/Minnesota/101/2024	9/6/24	0.16 ± 0.01 (1.5)	0.52 ± 0.14 (1.4)	0.14 ± 0.04 (1.2)	0.70 ± 0.20 (1.4)	1.05 ± 0.43 (0.8)	19433720

^*a*^ EC_50_, 50% effective concentration; IC_50_, 50% inhibitory concentration; NA, neuraminidase; "–" not applicable.

^*b*^ Subtype-specific median IC_50_s for NA inhibitors and EC_50_s for baloxavir were generated by testing indicated number of variant viruses collected in US during 2013–24 period (30).

^*c*^ This virus was sequenced flagged to test in NI assay as it contains V149I.

## DISCUSSION

Despite the high severity of the 2024–25 influenza season and the potential increase in the use of antiviral medications, drug-resistant viruses were detected at a low frequency in the US. Based on available data, most drug-resistant influenza viruses were collected from patients who were not exposed to antivirals. This indicates their ability to transmit from human to human and necessitates close monitoring of their spread (12, 14).

Emergence of oseltamivir-resistant influenza viruses of N1 subtype has been an ongoing public health concern (8). Oseltamivir is a cost-effective generic antiviral medication which is stocked by pharmacies for winter seasons (7, https://www.cdc.gov/flu/hcp/antivirals/summary-clinicians.html). NA substitution H275Y, the principal oseltamivir-resistance marker, was detected in <1% of A(H1N1)pdm09 viruses that circulated in the 2024–25 influenza season; this frequency was comparable to US national virologic surveillance in previous seasons (0.07% to 1.03%, Fig. 1B). To detect H275Y, WGS was performed by multiple laboratories (NIRCs and ISCs) that are strategically located across the US, representing geographically diverse or high-travel metropolitan areas and with regional catchments of specimens from other states. These activities boosted the number of viruses analyzed and improved data granularity at a state-level. Notably, WGS is indispensable in identifying diverse influenza strains (e.g., swine-origin vs seasonal virus), detecting known markers of resistance to antivirals with different mechanisms of action, and tracking the spread of drug-resistant mutants (39).

It was shown that viruses can acquire so called permissive NA mutations that can improve replicative fitness of oseltamivir-resistant viruses with H275Y (12, 14–16). One such NA mutation, N369K, became fixed in A(H1N1)pdm09 viruses circulating since 2013 (33). Another permissive mutation, R257K, has been infrequently seen in viruses circulating in recent years (https://nextstrain.org/seasonal-flu/h1n1pdm/na/6y?c=gt-NA_257&gmax=1418&gmin=9). This mutation was mainly associated with viruses from HA clade_subclade 5a.2a_C.1.9 and NA subclade D (previously known as subclade C.5.3.1). During the summer months of 2024 and the following winter season, most H275Y viruses from China belonged to this phylogenetic group and contained R257K. Our analysis revealed that this same phylogenetic group with R257K also caused the cluster of oseltamivir-resistant viruses in Japan in September 2024 (40). However, in the US and most other countries, H275Y viruses with R257K were only detected sporadically throughout the 2024–25 season. It is apparent that H275Y viruses, with or without R257K, are sufficiently fit to transmit from human to human but do not seem to have an advantage over wildtype viruses. Close monitoring of H275Y viruses is essential as they may gain such advantage by improving HA and NA functional balance, “hitchhiking,” or other mechanisms (9, 41).

The mutation S247N was also seen in NA subclade D, and while it was only detected at low frequencies in previous seasons, there was a drastic increase in its detections since summer of 2025, especially in Europe (36). Unlike viruses from NA subclade D with R257K, the viruses with S247N belonged to a different HA clade_subclade, 5a.2a.1_D.3.1.1. S247N was shown to have a moderate effect on NA activity and transmission in a guinea pig model (42, 43). However, the transmission study was conducted prior to the introduction of the permissive mutation N369K. By itself S247N confers only ~4-fold change in inhibition by oseltamivir, which is interpreted as normal inhibition (35). However, in combination with I223V, it confers ~13-fold RI by oseltamivir. S247N also potentiates the effect of H275Y that confers HRI by oseltamivir and peramivir (34, 42), and this observation was confirmed in the present study. On the other hand, the two other substitutions, S247R and S247G, do confer RI (15- to 37-fold) by oseltamivir (https://cdn.who.int/media/docs/default-source/influenza/laboratory---network/quality-assurance/human-nai-marker-table.pdf).

Unlike for A(H1N1)pdm09, oseltamivir-resistant A(H3N2) viruses have been detected very rarely by US national surveillance. During the high severity season of 2024–25, 3 such viruses were detected; 2 of them were collected from immunocompromised patients following oseltamivir treatment, while antiviral treatment data for the third case was not available. These viruses had common treatment-emergent NA substitutions, E119V or R292K, which were previously associated with compromised virus fitness. In B/Victoria lineage, A245D was identified in this study as the new NA marker of RI by zanamivir and peramivir.

When interpreting outcomes of NI assay testing, it is essential to keep in mind that this is not a phenotypic test as it does not assess antiviral effect on virus replication. While NA mutants of type A viruses with a >10-fold increase over the median IC_50_ are reported as displaying RI by oseltamivir, type B viruses are reported as normally inhibited by oseltamivir despite their median IC_50_ value being nearly 100-fold higher than type A.

Numerous naturally occurring and treatment-emergent PA substitutions have been shown to reduce baloxavir susceptibility in cell culture (6, https://cdn.who.int/media/docs/default-source/influenza/laboratory---network/quality-assurance/antiviral-susceptibility-influenza/pa-marker-who-table_28-11-2025_updated.pdf). To improve sequence-based assessment of baloxavir susceptibility, we implemented the augmented approach. PA sequences were screened not only for substitutions previously reported to confer >3-fold reduced susceptibility, but also at additional residues that may be involved in the drug’s binding (6, Table S2a). The latter is needed to improve existing knowledge on mutations occurring in nature that are “neutral” with respect to baloxavir susceptibility. Such information is valuable when drug susceptibility assessment is solely based on sequence analysis (e.g., viruses are not available to test).

PA substitution I38T is the most common baloxavir treatment-emergent mutation, and its detection in influenza A and B viruses is often interpreted as baloxavir-resistance (17, 18). In nature, I38T has rarely been seen, including viruses circulating in various animal species (44). No such mutants were detected by the CDC surveillance in prior seasons, except for 1 case in 2023 (6), who traveled from Japan where baloxavir is widely prescribed. In the 2024–25 season, 2 viruses with I38T were detected by the US national surveillance, and there was no known exposure to baloxavir. Like I38T, I38M is another rare natural variance and has also been reported as a treatment-emergent mutation (17, 44). A single virus with I38M was detected through the US state-level supplemental surveillance. The other detected PA substitutions, D27G, K34R, and A37T are examples of rare natural polymorphisms which have a mild effect (4- to 7-fold) on baloxavir susceptibility in cell culture. The influenza A viruses with E199D/G (secondary category) showed only 1- to 2-fold increase in EC_50_ compared to the respective sequence-matched control, which is interpreted as normal susceptibility. Therefore, they should be placed into the tertiary category based on the results of this study. Our results further emphasize that all the secondary category PA substitutions should be interpreted as “suspected reduced susceptibility” based on sequence-only analysis; when such virus isolates are available, phenotypic testing is required to confirm the phenotype (6, Table S2b).

Using cell culture-based assay IRINA, we showed that the baseline susceptibility remained similar across the last 3 seasons. However, due to a rather low provisional threshold (3-fold), it is desirable to annually test a subset of viruses to confirm that the type- and subtype-specific baselines have not changed due to virus evolution.

Evidence of limited human to human transmission of baloxavir-resistant viruses with I38T has been reported (45, 46). However, information on *in vitro* and *in vivo* replicative fitness of clinical virus isolates with I38T or I38M remains limited (45, 47–49). In this study, we showed that growth of viruses with I38T, but not I38M, was attenuated in 3 laboratory cell lines, albeit mildly.

The swine-origin variant viruses collected in the US during the reporting period contained adamantane-resistance conferring mutation, S31N. However, none had known markers of resistance to NA and PA inhibitors and were deemed susceptible to these antivirals. This assessment was supported by testing available isolates with antivirals using the subtype-specific baselines that were established in previous years (30).

The present study has several limitations. First, we do not have detailed information on how many prescriptions of antivirals were issued in the 2024–25 season compared to previous seasons. However, based on information regarding stocking patterns by pharmacies, it is safe to assume that 2 oral antivirals, oseltamivir and baloxavir, were stocked by pharmacies and that oseltamivir was used more often (7, 8). Another limitation is that information on antiviral exposure was limited to drug-resistance cases detected via US national surveillance. Third, it is also important to emphasize the arbitrary nature of cut-off values set for reporting drug susceptibility. Fourth, the assessment of replicative fitness of PA mutants was carried out in laboratory cell lines. However, it would be prudent to expand characterization by using differentiated primary human respiratory tract cells as well as to assess transmissibility of baloxavir-resistant viruses in animal models. Finally, we intentionally excluded the results from the CDC surveillance on high pathogenicity avian influenza viruses collected from humans in 2024–25 as data from that work is published before (50).

In conclusion, monitoring influenza antiviral susceptibility in the US has substantially improved by increasing WGS capacities and bioinformatic support at numerous public health laboratories. Data gathered through virologic surveillance gives much-needed information on drug susceptibility of circulating influenza viruses which are used to guide recommendations on antiviral use.

## Data Availability

All genome sequences and associated metadata supporting the findings of this study can be accessed through the GISAID data set identifier number: EPI_SET_260401bk and EPI_SET_260319dz and their corresponding persistent digital object identifier (https://epicov.org/epi3/epi_set/260401bk; https://epicov.org/epi3/epi_set/260319dz).

## SUPPLEMENTAL MATERIAL

Fig. S1 legend spectrum.01514-26-s0001.docx NAI susceptibility across the seasons.

Fig. S1 spectrum.01514-26-s0002.tif NAI susceptibility across the seasons

Supplemental tables spectrum.01514-26-s0003.docx Tables S1 to S7.

ASM does not own the copyrights to Supplemental Material that may be linked to, or accessed through, an article. The authors have granted ASM a non-exclusive, world-wide license to publish the Supplemental Material files. Please contact the corresponding author directly for reuse.

## References

[1] Hayden FG, Asher J, Cowling BJ, Hurt AC, Ikematsu H, Kuhlbusch K, Lemenuel-Diot A, Du Z, Meyers LA, Piedra PA, Takazono T, Yen HL, Monto AS. 2022. Reducing influenza virus transmission: the potential value of antiviral treatment. Clin Infect Dis 74:532–540. doi:10.1093/cid/ciab625 34245250 PMC8834654

[2] Uyeki TM, Hui DS, Zambon M, Wentworth DE, Monto AS. 2022. Influenza. Lancet 400:693–706. doi:10.1016/S0140-6736(22)00982-5 36030813 PMC9411419

[3] Bright RA, Medina M, Xu X, Perez-Oronoz G, Wallis TR, Davis XM, Povinelli L, Cox NJ, Klimov AI. 2005. Incidence of adamantane resistance among influenza A (H3N2) viruses isolated worldwide from 1994 to 2005: a cause for concern. Lancet 366:1175–1181. doi:10.1016/S0140-6736(05)67338-2 16198766

[4] Takashita E, Fujisaki S, Morita H, Nagata S, Miura H, Kishida N, Nakamura K, Shirakura M, Sato A, Akimoto M, Sugawara H, Mitamura K, Abe T, Ichikawa M, Yamazaki M, Watanabe S, Odagiri T, Hasegawa H, Influenza Virus Surveillance Group of Japan. 2026. Baloxavir susceptibility of seasonal influenza viruses during the first seven seasons of clinical use in Japan, 2017/18 to 2023/24. Euro Surveill 31:2500336. doi:10.2807/1560-7917.ES.2026.31.1.2500336 41508891 PMC12862291

[5] Centers for Disease Control and Prevention. 2006. High levels of adamantane resistance among influenza A (H3N2) viruses and interim guidelines for use of antiviral agents--United States, 2005-06 influenza season. MMWR Morb Mortal Wkly Rep 55:44–46. 16424859

[6] Patel MC, Nguyen HT, Mishin VP, Pascua PNQ, Champion C, Lopez-Esteva M, Merced-Morales A, Budd A, Kirby MK, Rambo-Martin B, Laplante J, Bateman A, St George K, Sullivan M, Steel J, Kondor RJ, Gubareva LV. 2025. Antiviral susceptibility monitoring: testing algorithm, methods, and findings for influenza season, 2023-2024. Antiviral Res 244:106299. doi:10.1016/j.antiviral.2025.106299 41083110 PMC12621569

[7] Beigel JH, Hayden FG. 2021. Influenza therapeutics in clinical practice—challenges and recent advances. Cold Spring Harb Perspect Med 11:a038463. doi:10.1101/cshperspect.a038463 32041763 PMC8015700

[8] Govorkova EA, Takashita E, Daniels RS, Fujisaki S, Presser LD, Patel MC, Huang W, Lackenby A, Nguyen HT, Pereyaslov D, Rattigan A, Brown SK, Samaan M, Subbarao K, Wong S, Wang D, Webby RJ, Yen HL, Zhang W, Meijer A, Gubareva LV. 2022. Global update on the susceptibilities of human influenza viruses to neuraminidase inhibitors and the cap-dependent endonuclease inhibitor baloxavir, 2018-2020. Antiviral Res 200:105281. doi:10.1016/j.antiviral.2022.105281 35292289 PMC9254721

[9] Holmes EC, Hurt AC, Dobbie Z, Clinch B, Oxford JS, Piedra PA. 2021. Understanding the impact of resistance to influenza antivirals. Clin Microbiol Rev 34:e00224-20. doi:10.1128/CMR.00224-20 33568554 PMC7950363

[10] Hurt AC. 2014. The epidemiology and spread of drug resistant human influenza viruses. Curr Opin Virol 8:22–29. doi:10.1016/j.coviro.2014.04.009 24866471

[11] Bloom JD, Gong LI, Baltimore D. 2010. Permissive secondary mutations enable the evolution of influenza oseltamivir resistance. Science 328:1272–1275. doi:10.1126/science.1187816 20522774 PMC2913718

[12] Hurt AC, Hardie K, Wilson NJ, Deng YM, Osbourn M, Leang SK, Lee RTC, Iannello P, Gehrig N, Shaw R, et al.. 2012. Characteristics of a widespread community cluster of H275Y oseltamivir-resistant A(H1N1)pdm09 influenza in Australia. J Infect Dis 206:148–157. doi:10.1093/infdis/jis337 22561367 PMC3379839

[13] Mohan T, Nguyen HT, Kniss K, Mishin VP, Merced-Morales AA, Laplante J, St George K, Blevins P, Chesnokov A, De La Cruz JA, Kondor R, Wentworth DE, Gubareva LV. 2021. Cluster of oseltamivir-resistant and hemagglutinin antigenically drifted influenza A(H1N1)pdm09 viruses, Texas, USA, January 2020. Emerg Infect Dis 27:1953–1957. doi:10.3201/eid2707.204593 34152954 PMC8237887

[14] Takashita E, Kiso M, Fujisaki S, Yokoyama M, Nakamura K, Shirakura M, Sato H, Odagiri T, Kawaoka Y, Tashiro M. 2015. Characterization of a large cluster of influenza A(H1N1)pdm09 viruses cross-resistant to oseltamivir and peramivir during the 2013-2014 influenza season in Japan. Antimicrob Agents Chemother 59:2607–2617. doi:10.1128/AAC.04836-14 25691635 PMC4394804

[15] Butler J, Hooper KA, Petrie S, Lee R, Maurer-Stroh S, Reh L, Guarnaccia T, Baas C, Xue L, Vitesnik S, Leang SK, McVernon J, Kelso A, Barr IG, McCaw JM, Bloom JD, Hurt AC. 2014. Estimating the fitness advantage conferred by permissive neuraminidase mutations in recent oseltamivir-resistant A(H1N1)pdm09 influenza viruses. PLoS Pathog 10:e1004065. doi:10.1371/journal.ppat.1004065 24699865 PMC3974874

[16] Bloom JD, Nayak JS, Baltimore D. 2011. A computational-experimental approach identifies mutations that enhance surface expression of an oseltamivir-resistant influenza neuraminidase. PLoS One 6:e22201. doi:10.1371/journal.pone.0022201 21799795 PMC3140507

[17] Ince WL, Smith FB, O’Rear JJ, Thomson M. 2020. Treatment-emergent influenza virus polymerase acidic substitutions independent of those at I38 associated with reduced baloxavir susceptibility and virus rebound in trials of baloxavir marboxil. J Infect Dis 222:957–961. doi:10.1093/infdis/jiaa164 32253432

[18] Omoto S, Speranzini V, Hashimoto T, Noshi T, Yamaguchi H, Kawai M, Kawaguchi K, Uehara T, Shishido T, Naito A, Cusack S. 2018. Characterization of influenza virus variants induced by treatment with the endonuclease inhibitor baloxavir marboxil. Sci Rep 8:9633. doi:10.1038/s41598-018-27890-4 29941893 PMC6018108

[19] Gubareva LV, Mishin VP, Patel MC, Chesnokov A, Nguyen HT, De La Cruz J, Spencer S, Campbell AP, Sinner M, Reid H, Garten R, Katz JM, Fry AM, Barnes J, Wentworth DE. 2019. Assessing baloxavir susceptibility of influenza viruses circulating in the United States during the 2016/17 and 2017/18 seasons. Euro Surveill 24:1800666. doi:10.2807/1560-7917.ES.2019.24.3.1800666 30670144 PMC6344838

[20] Jester B, Schwerzmann J, Mustaquim D, Aden T, Brammer L, Humes R, Shult P, Shahangian S, Gubareva L, Xu X, Miller J, Jernigan D. 2018. Mapping of the US domestic influenza virologic surveillance landscape. Emerg Infect Dis 24:1300–1306. doi:10.3201/eid2407.180028 29715078 PMC6038762

[21] Matrosovich M, Matrosovich T, Carr J, Roberts NA, Klenk HD. 2003. Overexpression of the alpha-2,6-sialyltransferase in MDCK cells increases influenza virus sensitivity to neuraminidase inhibitors. J Virol 77:8418–8425. doi:10.1128/jvi.77.15.8418-8425.2003 12857911 PMC165236

[22] Takada K, Kawakami C, Fan S, Chiba S, Zhong G, Gu C, Shimizu K, Takasaki S, Sakai-Tagawa Y, Lopes TJS, Dutta J, Khan Z, Kriti D, van Bakel H, Yamada S, Watanabe T, Imai M, Kawaoka Y. 2019. A humanized MDCK cell line for the efficient isolation and propagation of human influenza viruses. Nat Microbiol 4:1268–1273. doi:10.1038/s41564-019-0433-6 31036910 PMC12421904

[23] Zhou B, Wentworth DE. 2012. Influenza A virus molecular virology techniques. Methods Mol Biol 865:175–192. doi:10.1007/978-1-61779-621-0_11 22528160

[24] Shepard SS, Meno S, Bahl J, Wilson MM, Barnes J, Neuhaus E. 2016. Viral deep sequencing needs an adaptive approach: IRMA, the iterative refinement meta-assembler. BMC Genomics 17:708. doi:10.1186/s12864-016-3030-6 27595578 PMC5011931

[25] Shu Y, McCauley J. 2017. GISAID: global initiative on sharing all influenza data - from vision to reality. Euro Surveill 22:30494. doi:10.2807/1560-7917.ES.2017.22.13.30494 28382917 PMC5388101

[26] Aksamentov I, Roemer C, Hodcroft E, Neher R. 2021. Nextclade: clade assignment, mutation calling and quality control for viral genomes. J Open Source Softw 6:3773. doi:10.21105/joss.03773

[27] Neher RA, Huddleston J, Bedford T, Lewis NS, Harvey R, Galiano M, Byrne AMP, James S, Smith D, Łuksza M, Ruchnewitz D, Laessig M, Fujisaki S, Watanabe S, Hasegawa H, Hassell N, Wentworth DE, Kondor R, Deng YM, Dapat C, Subbarao K, Barr I. 2026. Nomenclature for tracking of genetic variation of seasonal influenza viruses. Influenza Other Respir Viruses 20:e70230. doi:10.1111/irv.70230 41688063 PMC12904685

[28] Okomo-Adhiambo M, Sleeman K, Lysén C, Nguyen HT, Xu X, Li Y, Klimov AI, Gubareva LV. 2013. Neuraminidase inhibitor susceptibility surveillance of influenza viruses circulating worldwide during the 2011 Southern Hemisphere season. Influenza Other Respir Viruses 7:645–658. doi:10.1111/irv.12113 23575174 PMC5781198

[29] Patel MC, Flanigan D, Feng C, Chesnokov A, Nguyen HT, Elal AA, Steel J, Kondor RJ, Wentworth DE, Gubareva LV, Mishin VP. 2022. An optimized cell-based assay to assess influenza virus replication by measuring neuraminidase activity and its applications for virological surveillance. Antiviral Res 208:105457. doi:10.1016/j.antiviral.2022.105457 36332755 PMC10149149

[30] Gao R, Pascua PNQ, Chesnokov A, Nguyen HT, Uyeki TM, Mishin VP, Zanders N, Cui D, Jang Y, Jones J, La Cruz JD, Di H, Davis CT, Gubareva LV. 2024. Antiviral susceptibility of swine-origin influenza A viruses isolated from humans, United States. Emerg Infect Dis 30:2303–2312. doi:10.3201/eid3011.240892 39378870 PMC11521183

[31] Hussain S, Meijer A, Govorkova EA, Dapat C, Gubareva LV, Barr IG, Brown SK, Daniels RS, Fujisaki S, Galiano M, Huang W, Kondor RJ, Lackenby A, Lewis N, Lo J, Nguyen HT, Patel MC, Pereyaslov D, Rattigan A, Samaan M, Wang D, Webby RJ, Yen HL, Zhang W, Takashita E. 2025. Global update on the susceptibilities of influenza viruses to neuraminidase inhibitors and the cap-dependent endonuclease inhibitor baloxavir, 2020-2023. Antiviral Res 241:106217. doi:10.1016/j.antiviral.2025.106217 40571063 PMC12391581

[32] Meijer A, Rebelo-de-Andrade H, Correia V, Besselaar T, Drager-Dayal R, Fry A, Gregory V, Gubareva L, Kageyama T, Lackenby A, Lo J, Odagiri T, Pereyaslov D, Siqueira MM, Takashita E, Tashiro M, Wang D, Wong S, Zhang W, Daniels RS, Hurt AC. 2014. Global update on the susceptibility of human influenza viruses to neuraminidase inhibitors, 2012-2013. Antiviral Res 110:31–41. doi:10.1016/j.antiviral.2014.07.001 25043638 PMC8851378

[33] Dai M, Du W, Martínez-Romero C, Leenders T, Wennekes T, Rimmelzwaan GF, van Kuppeveld FJM, Fouchier RAM, Garcia-Sastre A, de Vries E, de Haan CAM. 2021. Analysis of the evolution of pandemic influenza A(H1N1) virus neuraminidase reveals entanglement of different phenotypic characteristics. mBio 12:e00287-21. doi:10.1128/mBio.00287-21 33975931 PMC8262965

[34] Hurt AC, Lee RT, Leang SK, Cui L, Deng YM, Phuah SP, Caldwell N, Freeman K, Komadina N, Smith D, Speers D, Kelso A, Lin RT, Maurer-Stroh S, Barr IG. 2011. Increased detection in Australia and Singapore of a novel influenza A(H1N1)2009 variant with reduced oseltamivir and zanamivir sensitivity due to a S247N neuraminidase mutation. Euro Surveill 16:19884. doi:10.2807/ese.16.23.19884-en 21679678

[35] Patel MC, Nguyen HT, Pascua PNQ, Gao R, Steel J, Kondor RJ, Gubareva LV. 2024. Multicountry spread of influenza A(H1N1)pdm09 viruses with reduced oseltamivir inhibition, May 2023-February 2024. Emerg Infect Dis 30:1410–1415. doi:10.3201/eid3007.240480 38916572 PMC11210663

[36] Saubi N, Andrés C, Prats-Méndez I, González-Sánchez A, Davtyan A, Vásquez-Mercado R, Rando A, Nadal P, Esperalba J, Arnedo M, Vicente M, Balada E, Mendioroz J, Martín MC, García-Camuñas K, Vaz R, Najarro A, Bernalte S, Larrosa N, Antón A, SIVIC Infectious diseases sentinel network. 2025. Expansion of influenza A(H1N1)pdm09 NA:S247N viruses with reduced susceptibility to oseltamivir, Catalonia, Spain, and in Europe, July to October 2025. Euro Surveill 30:2500873. doi:10.2807/1560-7917.ES.2025.30.48.2500873 41346317 PMC12680917

[37] Okomo-Adhiambo M, Mishin VP, Sleeman K, Saguar E, Guevara H, Reisdorf E, Griesser RH, Spackman KJ, Mendenhall M, Carlos MP, Healey B, St George K, Laplante J, Aden T, Chester S, Xu X, Gubareva LV. 2016. Standardizing the influenza neuraminidase inhibition assay among United States public health laboratories conducting virological surveillance. Antiviral Res 128:28–35. doi:10.1016/j.antiviral.2016.01.009 26808479

[38] Naughtin M, Dyason JC, Mardy S, Sorn S, von Itzstein M, Buchy P. 2011. Neuraminidase inhibitor sensitivity and receptor-binding specificity of Cambodian clade 1 highly pathogenic H5N1 influenza virus. Antimicrob Agents Chemother 55:2004–2010. doi:10.1128/AAC.01773-10 21343450 PMC3088255

[39] Van Poelvoorde LAE, Saelens X, Thomas I, Roosens NH. 2020. Next-generation sequencing: an eye-opener for the surveillance of antiviral resistance in influenza. Trends Biotechnol 38:360–367. doi:10.1016/j.tibtech.2019.09.009 31810633

[40] Takashita E, Shimizu K, Usuku S, Senda R, Okubo I, Morita H, Nagata S, Fujisaki S, Miura H, Kishida N, Nakamura K, Shirakura M, Ichikawa M, Matsuzaki Y, Watanabe S, Takahashi Y, Hasegawa H. 2024. An outbreak of influenza A(H1N1)pdm09 antigenic variants exhibiting cross-resistance to oseltamivir and peramivir in an elementary school in Japan, September 2024. Euro Surveill 29:2400786. doi:10.2807/1560-7917.ES.2024.29.50.2400786 39668762 PMC11650510

[41] Wu WL, Lau S-Y, Chen Y, Wang G, Mok BW-Y, Wen X, Wang P, Song W, Lin T, Chan K-H, Yuen K-Y, Chen H. 2012. The 2008-2009 H1N1 influenza virus exhibits reduced susceptibility to antibody inhibition: Implications for the prevalence of oseltamivir resistant variant viruses. Antiviral Res 93:144–153. doi:10.1016/j.antiviral.2011.11.006 22138712 PMC3243069

[42] Pokorná J, Pachl P, Karlukova E, Hejdánek J, Řezáčová P, Machara A, Hudlický J, Konvalinka J, Kožíšek M. 2018. Kinetic, thermodynamic, and structural analysis of drug resistance mutations in neuraminidase from the 2009 pandemic influenza virus. Viruses 10:339. doi:10.3390/v10070339 29933553 PMC6071225

[43] Seibert CW, Rahmat S, Krammer F, Palese P, Bouvier NM. 2012. Efficient transmission of pandemic H1N1 influenza viruses with high-level oseltamivir resistance. J Virol 86:5386–5389. doi:10.1128/JVI.00151-12 22345446 PMC3347392

[44] Stevaert A, Dallocchio R, Dessì A, Pala N, Rogolino D, Sechi M, Naesens L. 2013. Mutational analysis of the binding pockets of the diketo acid inhibitor L-742,001 in the influenza virus PA endonuclease. J Virol 87:10524–10538. doi:10.1128/JVI.00832-13 23824822 PMC3807387

[45] Imai M, Yamashita M, Sakai-Tagawa Y, Iwatsuki-Horimoto K, Kiso M, Murakami J, Yasuhara A, Takada K, Ito M, Nakajima N, et al.. 2020. Influenza A variants with reduced susceptibility to baloxavir isolated from Japanese patients are fit and transmit through respiratory droplets. Nat Microbiol 5:27–33. doi:10.1038/s41564-019-0609-0 31768027 PMC13014278

[46] Takashita E, Ichikawa M, Morita H, Ogawa R, Fujisaki S, Shirakura M, Miura H, Nakamura K, Kishida N, Kuwahara T, Sugawara H, Sato A, Akimoto M, Mitamura K, Abe T, Yamazaki M, Watanabe S, Hasegawa H, Odagiri T. 2019. Human-to-human transmission of influenza A(H3N2) virus with reduced susceptibility to baloxavir, Japan, February 2019. Emerg Infect Dis 25:2108–2111. doi:10.3201/eid2511.190757 31436527 PMC6810216

[47] Chesnokov A, Patel MC, Mishin VP, De La Cruz JA, Lollis L, Nguyen HT, Dugan V, Wentworth DE, Gubareva LV. 2020. Replicative fitness of seasonal influenza A viruses with decreased susceptibility to baloxavir. J Infect Dis 221:367–371. doi:10.1093/infdis/jiz472 31541547 PMC8851376

[48] Koszalka P, George A, Dhanasekaran V, Hurt AC, Subbarao K. 2022. Effect of baloxavir and oseltamivir in combination on infection with influenza viruses with PA/I38T or PA/E23K substitutions in the ferret model. mBio 13:e0105622. doi:10.1128/mbio.01056-22 35938724 PMC9426601

[49] Lee LY, Zhou J, Koszalka P, Frise R, Farrukee R, Baba K, Miah S, Shishido T, Galiano M, Hashimoto T, Omoto S, Uehara T, Mifsud EJ, Collinson N, Kuhlbusch K, Clinch B, Wildum S, Barclay WS, Hurt AC. 2021. Evaluating the fitness of PA/I38T-substituted influenza A viruses with reduced baloxavir susceptibility in a competitive mixtures ferret model. PLoS Pathog 17:e1009527. doi:10.1371/journal.ppat.1009527 33956888 PMC8130947

[50] Pascua PNQ, Chesnokov AP, Nguyen HT, Champion C, Gao R, De La Cruz JA, Jang Y, Hatta Y, Guo Z, Uyeki TM, Di H, Stevens J, Davis CT, Gubareva LV. 2026. Antiviral susceptibility of clade 2.3.4.4b highly pathogenic avian influenza A(H5N1) viruses from humans in the United States, October 2024 to February 2025. Emerg Microbes Infect 15:2601372. doi:10.1080/22221751.2025.2601372 41392919 PMC12777753

