# Supplementary material for: Detection and characterization of antiviral-resistant viruses during the influenza season of 2024–25
Source: Microbiol Spectr. 2026 Aug 6;14(9):e01514-26. doi: 10.1128/spectrum.01514-26 (PMC13532263; doi:10.1128/spectrum.01514-26)
Supplement: Supplementary file 1 [file spectrum.01514-26-s0001.docx]

**Figure S1 legend**: Stability of type- or subtype-specific IC_50_ medians for NA inhibitors across different seasons. The IC_50_ medians for oseltamivir, zanamivir, peramivir, and laninamivir determined for 2016-25 seasons (excluding 2020-21 and 2021-22 seasons with very low number of influenza viruses in circulation) were compiled from CDC laboratory database and plotted for retrospective analysis. For each plot, y-axis is broken into two segments to accommodate higher median IC_50_s for B/Victoria viruses. In each NA inhibitor plot, the dashed line is shown as reference and derived by taking the average of type- or subtype-specific medians across indicated seasons.
