# Supplementary material for: Detection and characterization of antiviral-resistant viruses during the influenza season of 2024–25
Source: Microbiol Spectr. 2026 Aug 6;14(9):e01514-26. doi: 10.1128/spectrum.01514-26 (PMC13532263; doi:10.1128/spectrum.01514-26)
Supplement: Supplementary file 2 [file spectrum.01514-26-s0003.docx]

**Table S1: Summary of seasonal influenza positive specimens received and processed for three arms of surveillance system (US national, US supplemental in-state, and other countries), 2024-25 season**

| Surveillance laboratory | No. specimens | | | | | | | | | | | |
| --- | --- | --- | --- | --- | --- | --- | --- | --- | --- | --- | --- | --- |
|  | A(H1N1)pdm09 | | | A(H3N2) | | | B/Victoria | | | Total | | |
|  | Received | Sequenced | Propagated | Received | Sequenced | Propagated | Received | Sequenced | Propagated | Received | Sequenced | Propagated |
| **US National – 3 NIRCs**^a^ | | | | | | | | | | | | |
| California | 538 | 536 | 290 | 649 | 645 | 373 | 230 | 224 | 141 | 1417 | 1405 | 804 |
| New York | 962 | 955 | 647 | 878 | 875 | 572 | 491 | 491 | 376 | 2331 | 2321 | 1595 |
| Wisconsin | 648 | 638 | 328 | 884 | 875 | 491 | 207 | 200 | 134 | 1739 | 1713 | 953 |
| Total | **2148** | **2129** | **1265** | **2411** | **2395** | **1436** | **928** | **915** | **651** | **5487** | **5439** | **3352** |
| **US supplemental**^b^ **– New York NIRC (designated antiviral surveillance laboratory), California & Wisconsin NIRCs, and six ISCs** | | | | | | | | | | | | |
| **NIRCs** | | | | | | | | | | | | |
| New York  (Pyro + NGS) | 318 | 309 | 0 | 283 | 281 | 0 | 128 | 127 | 0 | 729 | 717 | 0 |
| California | 297 | 283 | 0 | 139 | 134 | 0 | 22 | 22 | 0 | 458 | 439 | 0 |
| Wisconsin | 232 | 223 | 0 | 140 | 137 | 0 | 20 | 18 | 0 | 392 | 378 | 0 |
| **ISCs**^c^ | | | | | | | | | | | | |
| Colorado | 339 | 316 | 0 | 365 | 342 | 0 | 63 | 55 | 0 | 767 | 713 | 0 |
| Florida | 259 | 232 | 0 | 282 | 245 | 0 | 15 | 12 | 0 | 556 | 489 | 0 |
| Hawaii | 184 | 177 | 0 | 72 | 71 | 0 | 76 | 76 | 0 | 332 | 324 | 0 |
| Massachusetts | 489 | 449 | 0 | 605 | 547 | 0 | 137 | 105 | 0 | 1231 | 1101 | 0 |
| Minnesota | 358 | 288 | 0 | 106 | 90 | 0 | 39 | 29 | 0 | 503 | 407 | 0 |
| Texas | 148 | 102 | 0 | 289 | 229 | 0 | 11 | 11 | 0 | 448 | 342 | 0 |
| **NIRCs + ISCs**  Total | **2624** | **2379** | **0** | **2281** | **2076** | **0** | **511** | **455** | **0** | **5416** | **4910** | **0** |
| **Non-US countries (n = 51)** | | | | | | | | | | | | |
| CDC laboratory | **952** | **886** | **592** | **675** | **625** | **448** | **690** | **576** | **372** | **2317** | **2087** | **1412** |

ISC, Influenza Sequencing Center; NGS, Next Generation Sequencing; NIRC, National Influenza Reference Center; Pyro, pyrosequencing

^a^The names of three NIRCs are: California Department of Public Health Viral and Rickettsial Disease Laboratory, New York State Department of Health (Wadsworth Center), and Wisconsin State Laboratory of Hygiene.

^b^US supplemental surveillance is only sequence-based. Therefore, viruses were not propagated for further testing.

^c^The names of six ISCs are: Colorado Department of Public Health and Environment, Florida Department of Public Health Laboratories, Hawaii State Department of Health, Massachusetts Department of Public Health, Minnesota Department of Health, and Texas Department of State Health Services.

**Table S2a: Inventory of PA amino acid residues monitored for sequence-based assessment of baloxavir susceptibility**

| **No.** | **Type A** | | **Type B** | | **Protein region**^a^**/ functional role** | **Rationale** | **References** |
| --- | --- | --- | --- | --- | --- | --- | --- |
|  | **Residue #** | **Conserved amino acid** | **Residue #** | **Conserved amino acid** |  |  |  |
| **Previously reported residues at which certain substitutions were implicated in reduced baloxavir susceptibility** | | | | | | | |
| 1 | 18 | E | 18 | K | N-ter/noncatalytic site | **E18G** in A(H1N1) RG virus conferred elevated baloxavir EC_50_ (10-fold). | Luo et al., 2023 |
| 2 | 23 | E | 23 | E | N-ter/noncatalytic site | Changes at this residue (**E23G**, **K**, **or R**) in type A viruses (RG or seasonal isolates) were shown to confer variably elevated baloxavir EC_50_ (1.8-19-fold);  **E23K** in B lineage RG viruses did not affect baloxavir EC_50_ (0.8-2.6-fold). | Omoto et al., 2018;  Gubareva et al., 2019;  Ince et al., 2020;  Takashita et al., 2020;  Abed et al., 2020;  Jones et al., 2022a, 2022b |
| 3 | 27 | D | 27 | D | N-ter/noncatalytic site | **D27G** in A(H1N1) RG virus conferred elevated baloxavir EC_50_ (12-fold). | Sun et al., 2026 |
| 4 | 28^b^ | L or P | 28 | P | N-ter/noncatalytic site | **L28P** in A(H3N2) RG virus conferred mild elevated baloxavir EC_50_ (2.6-fold);  This substitution in A(H3N2) seasonal isolates did not affect baloxavir EC_50_ (0.4-1.0-fold) vs sequence-matched controls. | Omoto et al., 2018;  Govorkova et al., 2022 |
| 5 | 34 | K | 34 | M | N-ter/active site | **K34R** in type A viruses conferred elevated baloxavir EC_50_ (3.7-5-fold) vs sequence-matched controls;  **M34I** in B virus did not affect baloxavir EC_50_ (0.6-fold). | Govorkova et al., 2022 |
| 6 | 36 | A | 36 | F | N-ter/noncatalytic site | **A36V** in type A RG viruses conferred elevated baloxavir EC_50_ (3.6-6-fold); **F36V** in B lineage RG virus did not affect baloxavir EC_50_ (0.8-fold). | Omoto et al., 2018 |
| 7 | 37 | A | 37 | N | N-ter/active site | **A37T** in type A viruses (RG or seasonal isolates; also, in A(H5N1)) conferred elevated baloxavir EC_50_ (5-8-fold). | Omoto et al., 2018;  Sato et al., 2021;  Nguyen et al., 2023 |
| 8 | 38 | I | 38 | I | N-ter/active site | **I38T** is recognized as primary molecular marker conferring resistance to baloxavir in both type A (11-614-fold elevated EC_50_) and B viruses (5-15-fold elevated EC_50_);  Other changes at this residue (**I38F**, **L**, **M**, **N**, or **S**) in type A viruses (RG or seasonal isolates; also, **I38F**/**M** in A(H5N1)) and (**I38F** or **M**) in B lineage RG viruses conferred variably elevated baloxavir EC_50_ (2.2-112-fold) and (1.7-8-fold), respectively. | Omoto et al., 2018;  Gubareva et al., 2019;  Jones et al., 2020;  Imai et al., 2020;  Hashimoto et al., 2021;  Govorkova et al., 2022;  Nguyen et al., 2023;  Taniguchi et al., 2024 |
| 9 | 119 | E | 120 | E | N-ter/metal binding | **E119D** in type A RG viruses conferred elevated baloxavir EC_50_ (5-6-fold); **E120D** in B lineage RG virus did not affect baloxavir EC_50_ (2.0-fold). | Omoto et al., 2018 |
| 10 | 198 | E | 199 | G | N-ter or linker/noncatalytic site | **E198K** in type A RG viruses conferred variably elevated baloxavir EC_50_ (1.8-6-fold);  **G199R** in B lineage RG virus did not affect baloxavir EC_50_ (2.0-fold). | Takizawa & Momose 2022;  Hickerson et al., 2022 |
| 11 | 199 | E | 200 | I | Linker/noncatalytic site | Changes at this residue (**E199D**, **G**, **or K**) in type A viruses (RG or seasonal isolates) conferred variably elevated baloxavir EC_50_ (0.5-7-fold). | Omoto et al., 2018;  Kiso et al., 2020;  Hashimoto et al., 2021;  Govorkova et al., 2022;  Hickerson et al., 2022  Takashita et al., 2023;  Takashita et al., 2025 |
| **Additional residues implicated in PA inhibitor binding via crystal structure or mutational analysis** | | | | | | | |
| 12 | 20 | A | 20 | T | N-ter/active site | Baloxavir makes van der Waals contact with Ala20 of type A;  **T20K** in influenza B virus was shown to confer elevated EC_50_ (7-fold). | Omoto et al., 2018;  Palmu et al., 2025 |
| 13 | 24 | Y | 24 | F | N-ter/active site | Baloxavir makes van der Waals contact with Tyr24 of type A;  **Y24C** in influenza A viruses were shown to confer variably elevated EC_50_ (2.0-5-fold). | Omoto et al., 2018  Patel et al., 2025  Takashita et al., 2026 |
| 14 | 41 | H | 41 | H | N-ter/metal binding | Side chain of His41 interacts with Mn^+2^ in the active center;  **H41A** in A(H1N1) RG virus was shown to confer elevated EC_50_ for L-742,001. | Stevaert et al., 2013;  Omoto et al., 2018 |
| 15 | 42 | L | 42 | L | N-ter/noncatalytic site | Leu42 is adjacent to the metal-chelating residue His41;  **L42T** in A(H1N1) RG virus was associated with mild elevated EC_50_ for L-742,001. | Stevaert et al., 2013 |
| 16 | 80 | E | 81 | E | N-ter/metal binding | Side chain of Glu80 or Glu81 interacts with Mn^+2^ in the active center. | Omoto et al., 2018 |
| 17 | 81 | G | 82 | G | N-ter/noncatalytic site | Gly81 is adjacent to the metal-chelating residue Glu80;  Changes at this residue (**G81 F**, **T or V**) in A(H1N1) RG viruses were shown to confer elevated EC_50_ for L-742,001. | Stevaert et al., 2013 |
| 18 | 84 | R | 85 | R | N-ter/noncatalytic site | Arg85 makes additional hydrophobic contact with baloxavir in type B but not in type A. | Omoto et al., 2018 |
| 19 | 106 | L | 107 | L | N-ter/noncatalytic site | Involve in hydrogen bond network for baloxavir binding;  **L106R** in type A RG viruses was associated with elevated baloxavir EC_50_. | Omoto et al., 2018;  Chen et al., 2024 |
| 20 | 107 | P | 108 | A | N-ter/noncatalytic site | Involve in hydrogen bond network for baloxavir binding. | Omoto et al., 2018 |
| 21 | 108 | D | 109 | D | N-ter/metal binding | Side chain of Asp108 or Asp109 interacts with Mn^+2^ in the active center. | Omoto et al., 2018 |
| 22 | 120 | I | 121 | V | N-ter/metal binding | Side chain of Iso120 or Val121 interacts with Mn^+2^ in the endonuclease active center;  **I120T** in A(H1N1) RG virus was shown to confer elevated EC_50_ for L-742,001. | Stevaert et al., 2013;  Omoto et al., 2018 |
| 23 | 122 | V | 123 | I | N-ter/noncatalytic site | Involve in hydrogen bond network for baloxavir binding;  **V122T** in A(H1N1) RG virus was associated with mild elevated EC_50_ for L-742,001;  **V122A** in influenza A virus was shown to confer elevated EC_50_ (9-11-fold). | Stevaert et al., 2013;  Omoto et al., 2018;  Patel et al., 2025 |
| 24 | 130 | Y | 131 | Y | N-ter/noncatalytic site | Involve in hydrogen bond network for baloxavir binding. | Omoto et al., 2018 |
| 25 | 134 | K | 135 | K | N-ter/noncatalytic site | Involve in hydrogen bond network for baloxavir binding. | Omoto et al., 2018 |

PA, polymerase acidic subunit; N-ter, N-terminal

^a^PA CEN domain for type A and type B viruses spans from amino acid residues 1-198 and 1-197, respectively (Omoto et al., 2018).

^b^The consensus amino acid at residue 28 is proline (P) and leucine (L) for A(H1N1)pdm09 and A(H3N2) virus backgrounds, respectively.

**Table S2b: Primary, secondary, and tertiary category PA substitutions: CDC-led sequence-based baloxavir susceptibility surveillance**

| **Category**   - ***Interpretation*** | **Common characteristics** | **Type A** | **Type B** |
| --- | --- | --- | --- |
| **Primary**   - *Reduced susceptibility* - *Phenotypic testing is not required, but helpful* | Primary treatment-emergent; associated with diminished virologic response in clinical settings;  Rarely as natural polymorphism;  Confers elevated baloxavir EC_50_ in cell culture (11-614-fold) | **I38T** | **I38T** |
|  | Treatment-emergent;  Rarely as natural polymorphisms;  Confers elevated baloxavir EC_50_ (4-112-fold) | I38 F, M, N, S | - |
| **Secondary**   - *Suspected reduced susceptibility* - *Phenotypic testing is required* | Most are natural polymorphisms;  Some emerged post baloxavir exposure or introduced for assessment;  Confers variably elevated baloxavir EC_50_ (<3-19-fold) | E18G | I38 F, M |
|  |  | E23 G, K, R | T20K |
|  |  | Y24*C* | - |
|  |  | D27G | - |
|  |  | K34R | - |
|  |  | A36V | - |
|  |  | A37T | - |
|  |  | I38L | - |
|  |  | L106R | - |
|  |  | E119D | - |
|  |  | V122A | - |
|  |  | E198K | - |
|  |  | E199 D, G, K | - |
| **Tertiary^a^**   - *Normal susceptibility* - *Phenotypic testing is not required, but helpful* | Natural polymorphisms;  No or little change in baloxavir EC_50_ (<3-fold) | A20 S, T, V | E23K |
|  |  | Y24 F, **H** | D27**N** |
|  |  | D27 **E**, **N** | M34 I, V |
|  |  | L28 M, P, **R**, V | F36 A, V |
|  |  | K34E | I38V |
|  |  | A36**T** | E120D |
|  |  | A37S | V121I |
|  |  | I38V | I123V |
|  |  | R84K | K134R |
|  |  | L106I | G199R |
|  |  | I120 L, V | I200**V** |
|  |  | V122 I, L | - |
|  |  | E198D | - |

PA, polymerase acidic subunit

^a^This category substitutions are compiled from references: Omoto et al., 2018, Gubareva et al., 2019, Hashimoto et al., 2021, Chen et al., 2024, Patel et al., 2025, and present study (shown in **bold**).

**Table S3: HA and NA clade _ subclade designations for H275Y viruses detected via CDC-led surveillance or submitted to GISAID by other US laboratories, 2024-25**

| **HA** | | **NA subclade** | **H275Y viruses in US state**^a^ **(n)** | | |
| --- | --- | --- | --- | --- | --- |
| **Clade** | **Subclade** |  | **CDC-led surveillance**^b^ | **Additional from GISAID**^c^ | **Total (%)** |
| 6B.1A.5a.2a | C.1.8 | C.5.3 | - | CA (1) | 1 (1.9) |
|  | C.1.9 | D | NC (1), NV (2), SD (1) | CA (1), MA (1)^d^, TN (1) | 7 (13) |
|  | C.1.9.2 | D | FL (1) | - | 1 (1.9) |
|  | C.1.9.3 | D.2 | FL (1) | MD (1), MI (2), OH (2) | 6 (11.1) |
| 6B.1A.5a.2a.1 | D.3.1 | D | - | MA (1) | 1 (1.9) |
|  | **D.3.1** | **D.1** | CA (1), FL (1), HI (2), MA (1), MN (2), OH (1), WI (2) | MA (1), MD (3), MI (6), NY (3), TX (1) | **24 (44.4)** |
|  | D.3.1 | D.2 | HI (1), NJ (1), WV (1) | MD (2), MI (2), NY (1), UT (1) | 9 (16.7) |
|  | D.3.1 | C.5.3 | MA (1) | MD (1) | 2 (3.7) |
|  | D | C.5.3 | - | MI (1) | 1 (1.9) |
|  | D.5 | C.5.3 | - | CA (1), MI (1) | 2 (3.7) |
| **Total** | | | 20 | 34 | 54 |

HA, hemagglutinin; NA, neuraminidase; GISAID, Global Initiative on Sharing All Influenza Data

^a^US states are listed using two-letter US postal abbreviations: CA (California), FL (Florida), HI (Hawaii), MA (Massachusetts), MD (Maryland), MI (Michigan), MN (Minnesota), NC (North Carolina), NJ (New Jersey), NV (Nevada), NY (New York), OH (Ohio), SD (South Dakota), TN (Tennessee), TX (Texas), UT (Utah), WI (Wisconsin), WV (West Virginia).

^b^A total of 24 H275Y viruses were detected via CDC-led surveillance (8 from national and 16 from supplemental in-state). Four H275Y viruses (3 detected via pyrosequencing at New York NIRC laboratory and 1 for which HA segment sequencing failed) were excluded from this analysis.

^c^NA sequences were accessed from GISAID on 27 February 2026.

^d^H275Y virus from Massachusetts contains R257K.

**Table S4: A(H1N1)pdm09 viruses with H275Y (n = 34) submitted to GISAID by other US laboratories, 2024-25**

| **Virus name** | **Date of collection (MM/DD/Yr)** | **GISAID Isolate Id (EPI_ISL_)** | **HA clade** | **HA subclade** | **NA subclade** | **Originating Lab** |
| --- | --- | --- | --- | --- | --- | --- |
| A/Baltimore/JH-859/2025 | XX/XX/2025 | 19821633 | 5a.2a.1 | D.3.1 | D.2 | Johns Hopkins School of Medicine |
| A/Baltimore/JH-653/2025 | XX/XX/2025 | 19787311 | 5a.2a.1 | D.3.1 | C.5.3 | Johns Hopkins School of Medicine |
| A/Baltimore/JH-1057/2025 | XX/XX/2025 | 19918888 | 5a.2a.1 | D.3.1 | D.2 | Johns Hopkins School of Medicine |
| A/Baltimore/JH-1182/2025 | XX/XX/2025 | 19919780 | 5a.2a.1 | D.3.1 | D.1 | Johns Hopkins School of Medicine |
| A/Baltimore/JH-1183/2025 | XX/XX/2025 | 19919781 | 5a.2a.1 | D.3.1 | D.1 | Johns Hopkins School of Medicine |
| A/Baltimore/JH-1184/2025 | XX/XX/2025 | 19919782 | 5a.2a.1 | D.3.1 | D.1 | Johns Hopkins School of Medicine |
| A/California/LACPHL-INF00856/2024 | 11/25/2024 | 19649966 | 5a.2a | C.1.8 | C.5.3 | Los Angeles County Public Health Laboratories, Lab Microbial Pathogen Submission Group |
| A/California/LACPHL-INF01006/2024 | 11/25/2024 | 19688225 | 5a.2a.1 | D.5 | C.5.3 | Los Angeles County Public Health Laboratories, Lab Microbial Pathogen Submission Group |
| A/California/LACPHL-INF01389/2025 | 1/1/2025 | 19777302 | 5a.2a | C.1.9 | D | Los Angeles County Public Health Laboratories, Lab Microbial Pathogen Submission Group |
| A/Maryland/MD-MDH-F-00167/2024 | 11/23/2024 | 19696608 | 5a.2a | C.1.9.3 | D.2 | Maryland Department of Health |
| A/Massachusetts/15641/2025 | 2/19/2025 | 19788784 | 5a.2a.1 | D.3.1 | D | US Air Force School of Aerospace Medicine |
| A/Massachusetts/IVY-G25Y78W6/2025 | 2/26/2025 | 19873411 | 5a.2a.1 | D.3.1 | D.1 | University of Michigan, Lauring Lab, Department of Microbiology and Immunology |
| A/Michigan/MIS-L385475116/2025 | 1/20/2025 | 20153413 | 5a.2a.1 | D.3.1 | D.1 | University of Michigan, Lauring Lab, Department of Microbiology and Immunology |
| A/Michigan/MIS-L387103753/2025 | 3/5/2025 | 20154218 | 5a.2a.1 | D.3.1 | D.1 | University of Michigan, Lauring Lab, Department of Microbiology and Immunology |
| A/Michigan/MIS-L388768714/2025 | 4/30/2025 | 20153800 | 5a.2a.1 | D.3.1 | D.2 | University of Michigan, Lauring Lab, Department of Microbiology and Immunology |
| A/Michigan/UM-10061579223/2025 | 1/2/2025 | 19699956 | 5a.2a.1 | D | C.5.3 | University of Michigan, Lauring Lab, Department of Microbiology and Immunology |
| A/Michigan/UM-10061617759/2025 | 1/5/2025 | 19699973 | 5a.2a | C.1.9.3 | D.2 | University of Michigan, Lauring Lab, Department of Microbiology and Immunology |
| A/Michigan/UM-10062129350/2025 | 2/1/2025 | 19765801 | 5a.2a.1 | D.3.1 | D.1 | University of Michigan, Lauring Lab, Department of Microbiology and Immunology |
| A/Michigan/UM-10062650833/2025 | 2/28/2025 | 19834621 | 5a.2a.1 | D.5 | C.5.3 | University of Michigan, Lauring Lab, Department of Microbiology and Immunology |
| A/Michigan/UM-10062676805/2025 | 3/3/2025 | 19834543 | 5a.2a.1 | D.3.1 | D.1 | University of Michigan, Lauring Lab, Department of Microbiology and Immunology |
| A/Michigan/UM-10062689116/2025 | 3/3/2025 | 19834581 | 5a.2a.1 | D.3.1 | D.1 | University of Michigan, Lauring Lab, Department of Microbiology and Immunology |
| A/Michigan/UM-10062741779/2025 | 3/6/2025 | 19834686 | 5a.2a.1 | D.3.1 | D.1 | University of Michigan, Lauring Lab, Department of Microbiology and Immunology |
| A/Michigan/UM-10063738210/2025 | 4/27/2025 | 19873463 | 5a.2a.1 | D.3.1 | D.2 | University of Michigan, Lauring Lab, Department of Microbiology and Immunology |
| A/Michigan/UMHM3304/2025 | 1/21/2025 | 19873286 | 5a.2a | C.1.9.3 | D.2 | University of Michigan, Lauring Lab, Department of Microbiology and Immunology |
| A/New York/PX18685/2025 | 2/16/2025 | 19843499 | 5a.2a.1 | D.3.1 | D.2 | Icahn School of Medicine at Mount Sinai |
| A/New York/PX20685/2025 | 3/30/2025 | 20100999 | 5a.2a.1 | D.3.1 | D.1 | Icahn School of Medicine at Mount Sinai |
| A/New York/PX20733/2025 | 4/2/2025 | 20101004 | 5a.2a.1 | D.3.1 | D.1 | Icahn School of Medicine at Mount Sinai |
| A/New York/PX21182/2025 | 4/2/2025 | 20101021 | 5a.2a.1 | D.3.1 | D.1 | Icahn School of Medicine at Mount Sinai |
| A/Ohio/IVY-G25Y96I2/2025 | 3/24/2025 | 19885267 | 5a.2a | C.1.9.3 | D.2 | University of Michigan, Lauring Lab, Department of Microbiology and Immunology |
| A/Ohio/PLMI-LYT8Y-00469/2025 | XX/XX/2025 | 20263477 | 5a.2a | C.1.9.3 | D.2 | Cleveland Clinic Foundation |
| A/Tennessee/IVY-G25Y71T2/2025 | 2/26/2025 | 19873264 | 5a.2a | C.1.9 | D | University of Michigan, Lauring Lab, Department of Microbiology and Immunology |
| A/Texas/16338/2025 | 5/19/2025 | 20197580 | 5a.2a.1 | D.3.1 | D.1 | US Air Force School of Aerospace Medicine |
| A/Utah/IVY-G25Y84W6/2025 | 3/7/2025 | 19873255 | 5a.2a.1 | D.3.1 | D.2 | University of Michigan, Lauring Lab, Department of Microbiology and Immunology |
| A/Massachusetts/ MA-BI_MGH-23684/2025 | 2/9/2025 | 19825509 | 5a.2a | C.1.9 | D | Broad Institute of Harvard and MIT |
| A/Massachusetts/MA-BI_MGH-23684/2025 | 2/9/2025 | 19825509 | 5a.2a | C.1.9 | D | Broad Institute of Harvard and MIT |

HA, hemagglutinin; NA, neuraminidase; GISAID, Global Initiative on Sharing All Influenza Data

**Table S5: HA and NA clade _ subclade designations for H275Y viruses collected in the US and other countries in 2024-25 season, GISAID data**

| **Country name**^a^ | **Total sequences in GISAID**^c^ | | **Viruses with H275Y** | | | | | |
| --- | --- | --- | --- | --- | --- | --- | --- | --- |
|  | **A(H1N1)pdm09 analyzed**^b^  **(% of major HA subclade)** | **With H275Y**  **(% of sequence analyzed)** | **HA clade** | **HA subclade** | **NA subclade** | **No. with H275Y only** | **No. with H275Y+R257K** | **Total** |
| Australia | 4643  (81% **D.3.1**) | 57 (1.2%) | 5a.2a | C.1.9 | D | 1 | - | 1 |
|  |  |  |  | C.1.9.3 | D.2 | 4 | - | 4 |
|  |  |  | **5a.2a.1** | **D.3.1** | **D.1** | 26 | - | 26 |
|  |  |  |  | **D.3.1** | **D.2** | 26 | - | 26 |
| Canada | 3277  (43% **D.3.1**; 40% C.1.9.3) | 18 (0.5%) | 5a.2a | C.1.9 | D | 1 | - | 1 |
|  |  |  |  | C.1.9.3 | D | 1 | - | 1 |
|  |  |  | **5a.2a.1** | **D.3.1** | **D.1** | 12 | 1 | 13 |
|  |  |  |  | D.3.1 | D.2 | 3 | - | 3 |
| China | 659  (57% **C.1.9**; 36% C.1.9.3) | 81 (12.3%) | **5a.2a** | **C.1.9** | **D** | 2 | 68 | 70 |
|  |  |  |  | C.1.9 | D.2 | 1 | - | 1 |
|  |  |  |  | C.1.9.3 | D | 3 | - | 3 |
|  |  |  |  | C.1.9.3 | D.2 | 6 | 1 | 7 |
| Chile | 989  (84% **D.3.1**) | 12 (1.2%) | **5a.2a.1** | D.3.1 | D.1 | 2 | - | 2 |
|  |  |  |  | **D.3.1** | **D.2** | 10 | - | 10 |
| Japan | 661  (52% **C.1.9.3**; 27% D.3.1) | 17 (2.6%) | **5a.2a** | C.1.9 | D | 1 | - | 1 |
|  |  |  |  | C.1.9.3 | D | 1 | - | 1 |
|  |  |  |  | **C.1.9.3** | **D.2** | 8 | 1 | 9 |
|  |  |  | 5a.2a.1 | D.3.1 | D.1 | 4 | - | 4 |
|  |  |  |  | D.3.1 | D.2 | 2 | - | 2 |
| Spain | 2678  (61% **C.1.9.3**) | 10 (0.4%) | **5a.2a** | C.1.9 | D | 1 | - | 1 |
|  |  |  |  | **C.1.9.3** | **D.2** | 6 | - | 6 |
|  |  |  | 5a.2a.1 | D.2 | C.5.3.2 | 1 | - | 1 |
|  |  |  |  | D.3.1 | D.1 | 2 | - | 2 |
| United Kingdom | 1414  (68% **C.1.9.3**) | 23 (1.6%) | **5a.2a** | C.1.9 | D | 1 |  | 1 |
|  |  |  |  | C.1.9.1 | D | 3 |  | 3 |
|  |  |  |  | **C.1.9.3** | **D.2** | 15 |  | 15 |
|  |  |  | 5a.2a.1 | D.3.1 | C.5.3 | 2 | - | 2 |
|  |  |  |  | D.3.1 | D.1 | 1 | - | 1 |
|  |  |  |  | D.5 | C.5.3 | 1 | - | 1 |
| United States | 9243  (59% **D.3.1**; 26% C.1.9.3) | 54 (0.6%) | 5a.2a | C.1.8 | C.5.3 | 1 |  | 1 |
|  |  |  |  | C.1.9 | D | 6 | 1 | 7 |
|  |  |  |  | C.1.9.2 | D | 1 |  | 1 |
|  |  |  |  | C.1.9.3 | D.2 | 6 |  | 6 |
|  |  |  | **5a.2a.1** | D.3.1 | D | 1 |  | 1 |
|  |  |  |  | **D.3.1** | **D.1** | 24 |  | 24 |
|  |  |  |  | D.3.1 | D.2 | 9 |  | 9 |
|  |  |  |  | D.3.1 | C.5.3 | 2 |  | 2 |
|  |  |  |  | D | C.5.3 | 1 |  | 1 |
|  |  |  |  | D.5 | C.5.3 | 2 |  | 2 |
|  |  |  |  | D.3.1 | D | 1 |  | 1 |

HA, hemagglutinin; NA, neuraminidase; GISAID, Global Initiative on Sharing All Influenza Data

^a^Only those countries which had ≥ 10 total sequences of A(H1N1)pdm09 viruses with H275Y submitted to GISAID were included in this analysis. Names of the countries are arranged in alphabetical order. Color coding is to indicate most common combination of HA clade_subclade and NA subclade.

^b^It is possible that some of the sequences of viruses submitted to GISAID may have come from specialized studies, such as clinical studies with antiviral treated patients, which may influence the rate of H275Y frequency.

^c^Overall, it is evident that most of H275Y viruses belonged to major circulating HA clade_subclade of A(H1N1)pdm09 viruses in each country. Additionally, except China (12.3%), rate of H275Y detection in other countries remained low (0.5-2.6%).

**Table S6: Neuraminidase inhibitor susceptibility assessment of available sequence-flagged viruses using NI assay, CDC-led surveillance 2024-25**

| (Sub)type or lineage | NA substitution^a^ | Virus name or number of viruses tested | IC_50_ nM (fold)^b^ | | | |
| --- | --- | --- | --- | --- | --- | --- |
|  |  |  | Oseltamivir | Zanamivir | Peramivir | Laninamivir |
| **NA substitutions associated with >10-fold increase in IC_50_** | | | | | | |
| A(H1N1)pdm09 | H275Y | n = 10 | 268.59 ± 32.18 (**814**) | 0.23 ± 0.03 (1) | 26.79 ± 1.80 (**447**) | 0.44 ± 0.04 (2) |
|  | H275Y, S247N | A/Belgium/0033/2025 | 1964.10 (**5952**) | 0.64 (4) | 49.54 (**826**) | 1.40 (7) |
|  | I223K | A/British Columbia/RV04868/2024 | 15.97 (**48**) | 0.73 (5) | 0.42 (7) | 0.67 (4) |
|  | I223V, S247N | n = 2 | 5.20, 4.72 (**16**, **14**) | 0.63, 0.57 (4, 4) | 0.32, 0.32 (5, 5) | 0.74, 0.80 (4, 4) |
|  | S247R | A/Pennsylvania/30/2025 | 9.20 (**28**)^c^ | 10.96 (**69**)^c^ | 13.11 (**219**)^c^ | 46.84 (**247**)^c^ |
| A(H3N2) | E119V | A/New York/17/2025 | 49.55 (**310**)^c^ | 1.37 (4)^c^ | 0.08 (1)^c^ | 0.43 (1)^c^ |
|  | E119V/E, R292K/R^d^ | A/Indiana/60/2024 - clone 1 – E119V | 137.99 (**142**) | 11.79 (**12**) | 0.42 (4) | 3.63 (6) |
|  |  | A/Indiana/60/2024 - clone 2 – R292K | 1813.49 (**1870**) | 21.00 (**22**) | 5.20 (**49**) | 7.92 (**14**) |
| B/Victoria | A245D | B/Minnesota/12/2025 | 48.90 (2)^e^ | 9.79 (**6.1**)^e^ | 2.75 (**5.7**)^e^ | 2.68 (3)^e^ |
| **NA substitutions associated with <10-fold increase in IC_50_** | | | | | | |
| A(H1N1)pdm09 | S247N | n = 26 | 1.49 ± 0.33 (5) | 0.19 ± 0.06 (1) | 0.37 ± 0.04 (6) | 0.61 ± 0.15 (3) |
|  | D199N | n = 2 | 1.45, 1.28 (4, 4) | 0.43, 0.37 (3, 2) | 0.08, 0.09 (1, 2) | 0.90, 0.33 (5, 2) |
|  | I223V | n = 2 | 1.36, 1.32 (4, 4) | 0.25, 0.24 (2, 2) | 0.07, 0.08 (1, 1) | 0.30, 0.32 (2, 2) |
|  | I436V | n =2 | 1.05, 3.04 (3, 9) | 0.42, 1.17 (3, 7) | 0.05, 0.21 (1, 4) | 0.26, 0.56 (1, 3) |
|  | I117T | A/Oklahoma/28/2025 | 1.81 (5) | 0.46 (3) | 0.04 (1) | 0.13 (1) |
|  | I427V | A/Nevada/103/2025 | 0.96 (3) | 0.26 (2) | 0.04 (1) | 0.15 (1) |
|  | T438A | A/Costa Rica/1717/2025 | 0.83 (3) | 0.28 (2) | 0.05 (1) | 0.17 (1) |
|  | T438S | A/Pennsylvania/281/2024 | 0.88 (3) | 0.30 (2) | 0.06 (1) | 0.17 (1) |
| A(H3N2) | Q391K | n = 2 | 0.18, 0.19 (1, 1) | 0.32, 0.29 (1, 1) | 0.07, 0.06 (1, 1) | 0.35, 0.40 (1, 1) |
|  | K249E | A/South Africa/3783/2025 | 0.20 (1) | 0.32 (1) | 0.07 (1) | 0.30 (1) |
| B/Victoria | T106I | B/Michigan/12/2025 | 31.95 (1) | 1.32 (1) | 1.09 (2) | 0.70 (1) |
|  | T106N | B/Minnesota/14/2025 | 33.76 (1) | 1.20 (1) | 0.64 (1) | 0.76 (1) |
|  | G247D | B/Vietnam/5072/2025 | 36.19 (1) | 6.26 (4) | 0.41 (1) | 1.17 (1) |
|  | K360E | B/Pennsylvania/03/2025 | 32.60 (1) | 1.11 (1) | 0.32 (1) | 0.66 (1) |

IC_50_, 50% inhibitory concentration; NA, neuraminidase; NI, NA inhibition

^a^Substitution is shown using type- or subtype-specific NA amino acid numbering system.

^b^Mean ± standard deviation of IC_50_s is shown when three or more viruses with indicated NA substitution were tested. If a single or two NA mutants were tested, individual IC_50_ value is shown. Fold change in IC_50_ was calculated versus subtype- or lineage-specific median IC_50_ generated by testing representative viruses that circulated during 2024-25 season (see Figure 2). For amino acid substitutions that conferred reduced or highly reduced inhibition by any of NA inhibitor, their respective folds are shown in bold.

^c^Fold change was also calculated versus IC_50_ of a respective NA sequence-matched control virus for an A(H1N1)pdm09 with S247R and A(H3N2) with E119V, which remained similar as determined using the median IC_50_.

^d^From this clinical specimen containing mix E119V/E and R292K/R, purified NA variants containing E119V or R292K were separated by limiting dilution. Purified clones were tested in NI assay and fold change was calculated versus IC_50_ of a wildtype clone.

^e^2-, 5.4-, 5.1-, and 4-fold increase over IC_50_s of NA sequence-matched control virus for oseltamivir, zanamivir, peramivir, and laninamivir, respectively.

**Table S7: Baloxavir susceptibility assessment of influenza viruses based on sequence analysis supplemented by IRINA, CDC-led surveillance 2024-25**

| Type/subtype or lineage | **PA sequence analysis** | | | | **Testing in IRINA** | |
| --- | --- | --- | --- | --- | --- | --- |
|  | PA substitution^a^ | US State (No. in each) | Other countries  (No. in each) | No. flagged viruses | No. isolates tested | Baloxavir EC_50_ nM (fold)^b^ |
| **Tertiary substitutions (normal susceptibility)** | | | | | | |
| A(H1N1)pdm09 | A20T | FL (1), MA (1), MN (1), NY (1), WI (1) | Colombia (1), Ghana (1), Argentina (1) | 8 | 3 | 0.93 ± 0.26 (1.2) |
|  | A20V | MA (1) | - | 1 | 0 | -^c^ |
|  | Y24F | NM (3) |  | 3 | 2 | 1.12, 1.28 (1.4, 1.6) |
|  | I38V | FL (1) | Cote d'Ivoire (1), Mali (1) | 3 | 0 | -^c^ |
|  | I120V | - | Brazil (1) | 1 | 0 | -^c^ |
|  | V122I | MA (1), NV (1), NY (1), TX (1) | Costa Rica (1) | 5 | 2 | 1.05, 0.85 (1.3, 1.1) |
| A(H3N2) | A20T | AZ (2), CA (1), DE (3), FL (2), KY (1), MA (18), MI (1), NM (13), NY (2), PA (2), UT (2), WV (1) | Bangladesh (1), Thailand (1) | 50 | 6 | 0.45 ± 0.09 (0.6) |
|  | L28M | TN (1) | - | 1 | 1 | 0.56 (0.8) |
|  | L28P | MN (1) | - | 1 | 1 | 0.97 (1.4) |
|  | R84K | MN (1), NV (1), SC (1), VA (1), WA (1) | - | 5 | 3 | 0.42 ± 0.09 (0.6) |
|  | I120V | TX (1) | - | 1 | 0 | -^c^ |
| B/Vic | M34I | PA (1) | Peru (1) | 2 | 1 | 1.68 (0.5) |
|  | V121I | CA (1), CO (12), GA (3), HI (1), IL (3), MA (1), MI (1), MN (2), MS (3), NC (2), NE (2), NV (1), NY (1), OR (2), WI (8) | Laos (1) | 44 | 15 | 2.24 ± 0.80 (0.7) |
|  | G199R | DC (1) | Nicaragua (1) | 2 | 2 | 2.87, 1.88 (0.8, 0.6) |
| **New Substitutions with unknown effect** | | | | | | |
| A(H1N1)pdm09 | D27E | CO (9), FL (4), HI (1), IA (3), ID (1), IL (2), KY (6), MA (7), MD (3), MI (1), MN (4), MO (6), MT (4), NC (1), NH (1), NV (6), NY (2), OH (1), PA (1), TN (4), TX (5), UT (1), WA (1), WI (30) | Canada (1) | 111 | 6 | 0.88 ± 0.36 (1) |
|  | D27N | FL (2), HI (1), ID (6), ND (1), NV (1), OR (1), PA (1), TX (1) | Argentina (1), Bolivia (1), Côte D'Ivoire (1), Jamaica (1), Nicaragua (1), Nigeria (5) | 24 | 4 | 0.94 ± 0.40 (1) |
|  | D27Y | CO (1) | - | 1 | 0 | - |
|  | P28S | VT (1) | - | 1 | 0 | - |
|  | A36T | MA (1) | - | 1 | 1 | 1.35 (1.7) |
|  | L42S | TX (1) | - | 1 | 0 | - |
| A(H3N2) | E18K | IA (1) | - | 1 | 0 | - |
|  | Y24H | IA (2), NE (4) | - | 6 | 6 | 1.42 ± 0.39 (2.0) |
|  | D27N | CO (1), FL (1), MA (4), NY (2) | - | 8 | 1 | 0.84 (1.2) |
|  | L28R | LA (1) | - | 1 | 1 | 1.23 (1.7) |
| B/Victoria | D27N | FL (1) | Cameroon (1) | 2 | 1 | 2.14 (0.6) |
|  | I200V | CO (4), MI (1), MT (1), NV (1), NM (4) | Bangladesh (1), Cameroon (1), Nicaragua (1) | 14 | 4 | 3.06 ± 0.74 (0.9) |

EC_50_, 50% effective concentration; IRINA, Influenza Replication Inhibition Neuraminidase-based Assay; PA, Polymerase acidic subunit

^a^PA CEN domain sequences of 11,008 influenza viruses (4656 A(H1N1)pdm09, 4679 A(H3N2), and 1673 B/Victoria; 9188 from US and 1820 from other countries) were screened to detect known or new substitution at 25 amino acid residues (see Table S2a). The substitutions at any of these residues were categorized into the four groups: primary, secondary, tertiary, and new substitution (Table S2b). IRINA testing results for viruses with primary or secondary category amino acid substitutions are shown in Table 2 and viruses with tertiary and new substitutions are shown in this Table. US states are listed using two-letter US postal abbreviations.

^b^Baloxavir susceptibility of available isolates of sequence-flagged viruses was assessed using a cell culture-based IRINA. Viruses were tested in at least 2 independent tests. Mean ± standard deviation of EC_50_s is shown when three or more viruses with indicated substitution were tested. Otherwise, mean EC_50_ value is shown. Fold changes in EC_50_s were calculated versus subtype- or lineage-specific median EC_50_s generated by testing representative viruses circulated during 2024-25 season (see Table 3). Testing outcome was interpreted based on provisional cut-off used previously by surveillance laboratories, according to which >3-fold increase in EC_50_ of a test virus versus reference EC_50_ is defined as reduced susceptibility to baloxavir.

^c^PA substitutions A20V, I38V, and I120V in influenza A viruses were shown previously not to affect baloxavir susceptibility by our laboratory (Patel et al., 2025, Gubareva et al., 2019).

**References:**

Abed, Y., Fage, C., Checkmahomed, L., Venable, M., Boivin, G., 2020. Characterization of contemporary influenza B recombinant viruses harboring mutations of reduced susceptibility to baloxavir marboxil, in vitro and in mice. Antivir Res 179, 104807.

Chen, D., Su, W., Choy, K.T., Chu, Y.S., Lin, C.H., Yen, H.L., 2024. High throughput profiling identified PA-L106R amino acid substitution in A(H1N1)pdm09 influenza virus that confers reduced susceptibility to baloxavir in vitro. Antivir Res 229, 105961.

Govorkova, E.A., Takashita, E., Daniels, R.S., Fujisaki, S., Presser, L.D., Patel, M.C., Huang, W., Lackenby, A., Nguyen, H.T., Pereyaslov, D., Rattigan, A., Brown, S.K., Samaan, M., Subbarao, K., Wong, S., Wang, D., Webby, R.J., Yen, H.L., Zhang, W., Meijer, A., Gubareva, L.V., 2022. Global update on the susceptibilities of human influenza viruses to neuraminidase inhibitors and the cap-dependent endonuclease inhibitor baloxavir, 2018-2020. Antivir Res 200, 105281.

Gubareva, L.V., Mishin, V.P., Patel, M.C., Chesnokov, A., Nguyen, H.T., De La Cruz, J., Spencer, S., Campbell, A.P., Sinner, M., Reid, H., Garten, R., Katz, J.M., Fry, A.M., Barnes, J., Wentworth, D.E., 2019. Assessing baloxavir susceptibility of influenza viruses circulating in the United States during the 2016/17 and 2017/18 seasons. Euro Surveill 24, 1800666.

Hashimoto, T., Baba, K., Inoue, K., Okane, M., Hata, S., Shishido, T., Naito, A., Wildum, S., Omoto, S., 2021. Comprehensive assessment of amino acid substitutions in the trimeric RNA polymerase complex of influenza A virus detected in clinical trials of baloxavir marboxil. Influenza Other Respir Viruses 15(3), 389-395.

Hickerson, B.T., Adams, S.E., Barman, S., Miller, L., Lugovtsev, V.Y., Webby, R.J., Ince, W.L., Donnelly, R.P., Ilyushina, N.A., 2022. Pleiotropic effects of influenza H1, H3, and B baloxavir-resistant substitutions on replication, sensitivity to baloxavir, and interferon expression. Antimicrob Agents Chemother 66(4), e0000922.

Imai, M., Yamashita, M., Sakai-Tagawa, Y., Iwatsuki-Horimoto, K., Kiso, M., Murakami, J., Yasuhara, A., Takada, K., Ito, M., Nakajima, N., Takahashi, K., Lopes, T.J.S., Dutta, J., Khan, Z., Kriti, D., van Bakel, H., Tokita, A., Hagiwara, H., Izumida, N., Kuroki, H., Nishino, T., Wada, N., Koga, M., Adachi, E., Jubishi, D., Hasegawa, H., Kawaoka, Y., 2020. Influenza A variants with reduced susceptibility to baloxavir isolated from Japanese patients are fit and transmit through respiratory droplets. Nat Microbiol 5, 27-33.

Ince, W.L., Smith, F.B., O'Rear, J.J., Thomson, M., 2020. Treatment-Emergent Influenza Virus Polymerase Acidic Substitutions Independent of Those at I38 Associated With Reduced Baloxavir Susceptibility and Virus Rebound in Trials of Baloxavir Marboxil. J Infect Dis 222, 957-961.

Jones, J.C., Pascua, P.N.Q., Fabrizio, T.P., Marathe, B.M., Seiler, P., Barman, S., Webby, R.J., Webster, R.G., Govorkova, E.A., 2020. Influenza A and B viruses with reduced baloxavir susceptibility display attenuated in vitro fitness but retain ferret transmissibility. Proc Natl Acad Sci U S A 117, 8593-8601.

Jones, J.C., Zagribelnyy, B., Pascua, P.N.Q., Bezrukov, D.S., Barman, S., Okda, F., Webby, R.J., Ivanenkov, Y.A., Govorkova, E.A., 2022a. Influenza A virus polymerase acidic protein E23G/K substitutions weaken key baloxavir drug-binding contacts with minimal impact on replication and transmission. PLoS Pathog 18, e1010698.

Jones, J.C., Rovito, S.W., Penaflor, M.K., Webby, R.J., Govorkova, E.A., 2022b. Influenza A virus polymerase acidic protein E23R substitution is a marker of reduced susceptibility to baloxavir. Antivir Res 204, 105369.

Kiso, M., Yamayoshi, S., Murakami, J., Kawaoka, Y., 2020. Baloxavir Marboxil Treatment of Nude Mice Infected with Influenza A Virus. J Infect Dis 221, 1699-1702.

Luo, D., Ye, Q., Li, R.T., Zhou, H.Y., Guo, J.J., Zhao, S.Q., Zhang, S., Jiang, T., Deng, Y.Q., Qin, C.F., 2023. PA-E18G substitution in influenza A virus confers resistance to ZX-7101, a cap-dependent endonuclease inhibitor. Virol Sin 38(4), 559-567.

Nguyen, H.T., Chesnokov, A., De La Cruz, J., Pascua, P.N.Q., Mishin, V.P., Jang, Y., Jones, J., Di, H., Ivashchenko, A.A., Killian, M.L., Torchetti, M.K., Lantz, K., Wentworth, D.E., Davis, C.T., Ivachtchenko, A.V., Gubareva, L.V., 2023. Antiviral susceptibility of clade 2.3.4.4b highly pathogenic avian influenza A(H5N1) viruses isolated from birds and mammals in the United States, 2022. Antivir Res 217, 105679.

Omoto, S., Speranzini, V., Hashimoto, T., Noshi, T., Yamaguchi, H., Kawai, M., Kawaguchi, K., Uehara, T., Shishido, T., Naito, A., Cusack, S., 2018. Characterization of influenza virus variants induced by treatment with the endonuclease inhibitor baloxavir marboxil. Sci Rep 8, 9633.

Palmu, S., Pillay-Ramaya, L., Baker, J., Kocsis, K., Kanwar, M., Berisha, E., Wildum, S., Macutkiewicz, L.B., Parra M.M., 2025. A phase 3 safety and efficacy study of baloxavir marboxil in children less than 1 year old with suspected or confirmed influenza. Pediatr Infect Dis J, PMID 40279637.

Sato, M., Takashita, E., Katayose, M., Nemoto, K., Sakai, N., Fujisaki, S., Hashimoto, K., Hosoya, M., 2021. Detection of Variants with Reduced Baloxavir Marboxil and Oseltamivir Susceptibility in Children with Influenza A During the 2019-2020 Influenza Season. J Infect Dis 224, 1735-1741.

Patel, M.C., Nguyen, H.T., Mishin, V.P., Pascua, P.N.Q., Champion, C., Lopez-Esteva, M., Merced-Morales, A., Budd, A., Kirby, M.K., Rambo-Martin, B., Laplante, J., Bateman, A., St George, K., Sullivan, M., Steel, J., Kondor, R.J., Gubareva, L.V., 2025. Antiviral susceptibility monitoring: testing algorithm, methods, and findings for influenza season, 2023-2024. Antiviral Res 244:106299.

Stevaert, A., Dallocchio, R., Dessi, A., Pala, N., Rogolino, D., Sechi, M., Naesens, L., 2013. Mutational analysis of the binding pockets of the diketo acid inhibitor L-742,001 in the influenza virus PA endonuclease. J Virol 87, 10524-10538.

Sun, B., Sun, Y., Wang, H., Tang, X., Tang, W., Mi, Y., Shen, Z., Xue, Q., Lu, Y., Zhao, X., Ai, J., Lu, J., Zhang, W., 2026. Emergence of the novel PA-D27G mutation conferring reduced baloxavir susceptibility in influenza A viruses circulating in China, 2018-2025. Emerg Microbes Infect 15:2620222.

Takashita, E., Abe, T., Morita, H., Nagata, S., Fujisaki, S., Miura, H., Shirakura, M., Kishida, N., Nakamura, K., Kuwahara, T., Mitamura, K., Ichikawa, M., Yamazaki, M., Watanabe, S., Hasegawa, H., Influenza Virus Surveillance Group of Japan., 2020. Influenza A(H1N1)pdm09 virus exhibiting reduced susceptibility to baloxavir due to a PA E23K substitution detected from a child without baloxavir treatment. Antivir Res 180, 104828.

Takashita, E., Fujisaki, S., Morita, H., Nagata, S., Miura, H., Matsuura, Y., Yamamoto, S., Chiba, S., Inoue, Y., Minami, I., Yoshikawa, S., Yamazaki, S., Kishida, N., Nakamura, K., Shirakura, M., Watanabe, S., Hasegawa, H., 2023. A community cluster of influenza A(H3N2) virus infection with reduced susceptibility to baloxavir due to a PA E199G substitution in Japan, February to March 2023. Euro Surveill 28(39), 2300501.

Takashita, E., Yasui, Y., Ikegaya, A., Saka, K., Maeshiro, N., Morita, H., Nagata, S., Fujisaki, S., Miura, H., Kishida, N., Nakamura, K., Shirakura, M., Watanabe, S., Hasegawa, H., The Influenza Virus Surveillance Group of Japan, 2025. Impact of the polymerase acidic protein E199K substitution in influenza A viruses on baloxavir susceptibility. Antivir Res, 106173, PMID 40324596.

Takashita, E., Fujisaki, S., Morita, H., Nagata, S., Miura, H., Kishida, N., Nakamura, K., Shirakura, M., Sato, A., Akimoto, M., Sugawara, H., Mitamura, K., Abe, T., Ichikawa, M., Yamazaki, M., Watanabe, S., Odagiri, T., Hasegawa, H., Influenza Virus Surveillance Group of Japan. 2026. Baloxavir susceptibility of seasonal influenza viruses during the first seven seasons of clinical use in Japan, 2017/18 to 2023/24. Euro Surveill 31:2500336.

Taniguchi, K., Noshi, T., Omoto, S., Sato, A., Shishido, T., Matsuno, K., Okamatsu, M., Krauss, S., Webby, R.J., Sakoda, Y., Kida, H., 2024. The impact of PA/I38 substitutions and PA polymorphisms on the susceptibility of zoonotic influenza A viruses to baloxavir. Arch Virol 169, 29.

Takizawa, N., Momose, F., 2022. A novel E198K substitution in the PA gene of influenza A virus with reduced susceptibility to baloxavir acid. Arch Virol 167, 1565-1570.
